# Oil degradation potential of microbial communities in water and sediment of Baltic Sea coastal area

**DOI:** 10.1371/journal.pone.0218834

**Published:** 2019-07-02

**Authors:** Hanna Miettinen, Malin Bomberg, Mari Nyyssönen, Anna Reunamo, Kirsten S. Jørgensen, Minna Vikman

**Affiliations:** 1 Solutions for Natural Resources and Environment, VTT Technical Research Centre of Finland Ltd, VTT, Finland; 2 Marine Research Centre, Finnish Environment Institute SYKE, Helsinki, Finland; Stazione Zoologica Anton Dohrn, ITALY

## Abstract

Two long-term potentially oil exposed Baltic Sea coastal sites near old oil refineries and harbours were compared to nearby less exposed sites in terms of bacterial, archaeal and fungal microbiomes and oil degradation potential. The bacterial, archaeal and fungal diversities were similar in oil exposed and less exposed sampling sites based on bacterial and archaeal 16S rRNA gene and fungal 5.8S rRNA gene amplicon sequencing from both DNA and RNA fractions. The number of genes participating in alkane degradation (*alk*B) or PAH-ring hydroxylation (PAH–RHDα) were detected by qPCR in all water and sediment samples. These numbers correlated with the number of bacterial 16S rRNA gene copies in sediment samples but not with the concentration of petroleum hydrocarbons or PAHs. This indicates that both the clean and the more polluted sites at the Baltic Sea coastal areas have a potential for petroleum hydrocarbon degradation. The active community (based on RNA) of the coastal Baltic Sea water differed largely from the total community (based on DNA). The most noticeable difference was seen in the bacterial community in the water samples were the active community was dominated by Cyanobacteria and Proteobacteria whereas in total bacterial community Actinobacteria was the most abundant phylum. The abundance, richness and diversity of Fungi present in water and sediment samples was in general lower than that of Bacteria and Archaea. Furthermore, the sampling location influenced the fungal community composition, whereas the bacterial and archaeal communities were not influenced. This may indicate that the fungal species that are adapted to the Baltic Sea environments are few and that Fungi are potentially more vulnerable to or affected by the Baltic Sea conditions than Bacteria and Archaea.

## Introduction

The Baltic Sea is a sensitive sea area as designated by the International Maritime Organization (IMO) in 2005. Sensitivity is due to several factors such as the low volumes of water, the mean depth of the Baltic Sea being only 54 m, and the slow exchange of water that occurs only through the narrow straits of Denmark to the North Sea and the Atlantic Ocean. In addition, the vertical thermal and salinity stratification as well as the low average temperature connected to the sea ice formation during winter times in the north parts of the Baltic Sea increase the vulnerability. The Baltic Sea water system is brackish and has a specific ecosystem including a great number of islands, wetlands and lagoons. Simultaneously, the Baltic Sea is one of the busiest waterways in the world. Considering the almost 300 000 yearly ship visits in the ports of the Baltic Sea region [1] the maritime traffic is very dense. Two thirds of the ships in the area are cargo ships of which a little less than half are tankers [1]. The yearly oil and chemical product transportation of over 300 Mt [1, 2] in the Baltic Sea comprises a significant risk of oil and chemical spills. Besides the traditional maritime transportation and fisheries, human activities such as offshore wind farms, underwater cables and pipelines, oil and gas exploitation and recreational activities in the Baltic Sea area have increased during past decades. Overall, the large catchment area of the Baltic Sea with diverse human activities cause eutrophication and result in a supply of polluting substances that degrade and modify the marine Baltic Sea ecosystems.

Specific locations in the Baltic Sea coastal area, such as ports and vicinities of industrial facilities that utilise or refine oil compounds, have suffered from frequent small oil discharges and spills for decades. In these places the benthic microbiomes may have adapted to this continuous or intermittent exposure of oil discharge and are potentially more effective in oil biodegradation than microbiomes in more pristine sites [3]. Microbial oil degradation is based on the ability of microorganism to utilise petroleum hydrocarbons for cell growth and energy. Research of marine oil biodegradation has focused largely on the degradation ability of oceanic Bacteria, reviewed e.g. by [4, 5]. However, many Fungi of marine origin are also capable of effective oil-degradation [6–9]. The role of Archaea in oil degradation in oceans in not fully understood [10] but there are signs that some Archaea such as Bathyarchaeota (formerly Miscellaneous Crenarchaeotal Group, MCG) and Halobacteriaceae may play a role in oil degradation [11, 12]. Archaeal oil degradation has not been as comprehensively studied as the bacterial oil degradation ability and there may still be marine taxa that have oil degradation capability to be detected in the future. The overall degradation of oil demands interactions and cooperation of many different species having complementary catabolic pathways [4] as oil comprises of several different types of compounds such as alkanes, cycloalkanes, alkenes, polycyclic aromatic hydrocarbons and asphaltenes. In addition, the ability of a microbial community to disperse and increase the bioavailability of petroleum hydrocarbons is needed in oil biodegradation.

Oil degradation potential of an unknown microbial community can be assessed based on the amounts of the genes involved in the oil degradation pathways. A well characterised aerobic short- and medium-chain-length alkane degradation pathway includes as its first enzyme an integral-membrane non-heme diiron monooxygenase, that is encoded by the gene *alk*B [13]. This gene has been detected especially from Proteobacteria, Actinobacteria, Bacteroidetes and Spirochaetes [14]. Another alkane hydroxylase involved in degradation of alkanes, is a cytochrome P450 CYP153 family [15] that has been detected in Proteobacteria, Actinobacteria and Planctomycetes but less frequently than the *alk*B gene [14]. Aromatic compound biodegradation in aerobic conditions commonly starts with the incorporation of molecular oxygen into the aromatic nucleus by the enzyme system RHD, a multicomponent aromatic ring-hydroxylating -dioxygenase [16]. This enzyme system composes of large α and small β subunits [17]. The large subunit (PAH-RHDα) is found in many Gram-negative Proteobacteria. Gram-positive Bacteria possess similar enzymatic system, but with different PAH-substrate specificity [16].

The objective of this study was to compare two long-term oil exposed Baltic Sea coastal sites to nearby less exposed sites in terms of bacterial, archaeal, and fungal microbiomes by amplicon sequencing. Oil degradation potential was determined by quantitative PCR (qPCR) of oil degradation genes *alk*B and PAH ring hydroxylating deoxygenase genes (PAH–RHDα) to test the hypothesis of higher oil degradation potential in oil polluted sites. Additionally, our aim was to clarify the differences between total communities (based on DNA) and active communities (based on RNA) in the water phase and total communities in the sea floor surface sediment. Extracellular DNA in marine sediments may affect the estimates of both microbial diversity and the abundance of for example the oil degradation potential of the living community. For this reason, the sediment communities were studied both from the total DNA and from the intracellular DNA (DNase treated samples).

## Materials and methods

### Site description and sample collection

**Fig 1:**
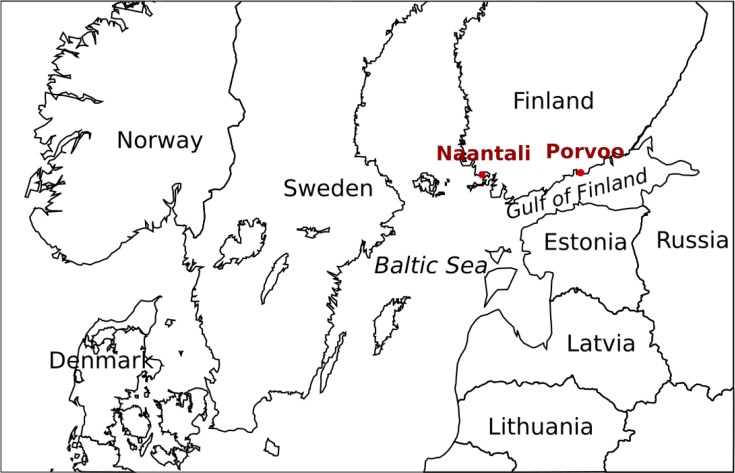
Sampling locations in the northern Baltic Sea. Sampling locations Naantali and Porvoo marked with red circles.

This study focused on two marine locations situated near oil refineries established in the 1950s and 1960s that are still in active use. The studied locations are situated in the Porvoo area on the coast of the Gulf of Finland, and in the Naantali area on the Finnish Archipelago Sea (Fig 1). The Porvoo industrial site is the biggest oil refinery and chemical industry centre in the Nordic countries. Both locations have also an active harbour. These sites were chosen because they have experienced oil discharges over the years and at the moment the environmental permits allow discharge of low amounts of oil. The precise sampling sites were chosen to be near waste and cooling water outlets from the refineries and other industries (Table 1) approximately 50 m from the shore. In addition, we studied two marine reference sites less impacted by oil, located at a distance of 5 km from the Porvoo refinery and 8 km away from the Naantali refinery. The sampling sites did not involve endangered or protected species. Permission for the near refinery samplings was received from the Neste Oyj and for the reference sites no permission was required.

**Table 1:** The Baltic Sea sampling locations, codes, sample types, coordinates, dates, depths and temperatures.

Site	Code	Sample type	Coordinates Lat/Long	Sampling date	Depth m	T °C	Other comments
Naantali	PP	Water	60.446150, 22.100520	19.11.2015	0.5	6.9	Near refinery treated waste water outlet, around 50 m from the shore
		Sediment			5		
	300	Water	60.486520, 21.968020	19.11.2015	0.5	6.7	Around 8 km from the refinery area
		Sediment			18.5		
Porvoo	Q	Water	60.309450, 25.554800	28.10.2015	0.5	9.2	Near refinery and petrochemical industry treated waste water outlet, around 50 m from the shore
		Sediment			20		
	D	Water	60.299090, 25.548280	28.10.2015	0.5	11.2	Near refinery cooling water and petrochemical industry treated waste water outlet, around 50 m from the shore
		Sediment			18		
	B	Water	60.264060, 25.555740	28.10.2015	0.5	9.1	Around 5 km from the refinery area
		Sediment			35		

Samples were collected from Porvoo and Naantali on the October 28^th^ and November 19^th^ 2015, respectively. Water samples (Table 1) were collected with a 1L Limnos sampler from 0.5 m depth in to new disposable unused plastic 10 L containers. Sediment was collected using an Ekman sampler from the uppermost 5 cm of the sediment layer and placed in to new disposable unused plastic buckets. Three parallel water samples of 180 mL (sampling points Q, D) and 240 mL (sampling points B, 300, PP) were collected on 0.22 μm pore-size Sterivex^TM^ filter units (Millipore Corporation, USA) using 60 mL disposable plastic syringes. Samples for RNA extraction were kept cool (+4°C) and filtered within 8 hours of sampling, placed in sterile 50 mL test tubes and immediately frozen in liquid nitrogen or on dry ice. Water samples for DNA extraction were also kept cool (+4°C) during transportation and storage until filtration. DNA samples from Porvoo were filtered within 48 h of sampling and Naantali samples within 12 h of sampling. From each sampling location, three replicates of 200 mL water samples were filtered on 0.22 μm pore-size cellulose acetate bottle-top filters (Corning, NY, USA) using mild vacuum suction in a laminar flow hood. The filter membranes were aseptically removed from the filter units with sterile scalpels and frozen at -80°C in sterile 50 mL plastic test tubes.

For chemical measurements, four replicate 50 mL water samples were collected in sterile plastic test tubes and frozen at -20°C until analyses. The bulk sediment samples were mixed thoroughly by manual stirring before three replicate 40 mL samples were collected into sterile plastic test tubes and stored at -20°C until use.

### Chemical analysis

Carbon, nitrogen and sulphur contents of the sediments were analysed using a Flash 2000 EA CHNS-O (Thermo Fisher Scientific, USA) -analyser with three replicate samples. Sediment samples were dried at +105°C for 24 hours and milled before analysis. Organic matter contents were determined by subtracting the residue of a known volume of sample after incineration at 550°C (Nabertherm GmbH oven, Germany) from the total dry solid content of the same sample. Organic matter contents were analysed using three replicate samples.

The following chemical water analyses were performed by Ramboll Analytics. The concentrations of total N, ammonium NH_4_-N, phosphate PO_4_-P and total P in sea water samples were analysed spectroscopically. The concentrations of nitrate NO_3_-N, nitrite NO_2_-N and sulphate SO_4_^2-^ were analysed by ion chromatography (IC), chloride concentration by measuring conductivity and total Fe by inductively coupled plasma mass spectrometry (ICP-MS). The measurement uncertainties of chemical water analyses are shown in the Table 2. The samples were analysed for the concentrations of petroleum hydrocarbons (sum between C_10_–C_40_) and polycyclic aromatic hydrocarbons (PAHs) as described in Reunamo et al. [18]. Two parallel samples were measured from each site.

**Table 2:** Concentrations of Cl^-^, SO42-, total N, NO_3_-N, NO_2_-N, PO_4_-P, total Fe, salinity, petroleum hydrocarbon and total PAHs in marine water samples. NH_4_-N < 20 μg L^-1^ in all samples. Salinity results of Porvoo extracted from [19] and of Naantali from [20].

Site	Cl^-^ mg L^-1^	SO_4_^2-^ mg L^-1^	Total N μg L^-1^	NO_3_-N μg L^-1^	NO_2_-N μg L^-1^	Total P μg L^-1^	PO_4_-P μg L^-1^	Total Fe μg L^-1^	Salinity PSU	C_10_-C_40_ μg L^-1^	Total PAHs^a^ μg L^-1^
Porvoo Q	2700	420	310	<4.0	<2.0	26	18	24	5.0	35	0.005
Porvoo D	2900	430	310	<4.0	<2.0	26	17	87	5.0	38	0.010
Porvoo B	2600	430	280	17	2.1	27	15	<10	5.1	37	0.010
Naantali PP	2700	400	680	<4.0	<2.0	43	11	310	5.7	23	0.140
Naantali 300	2600	410	300	11	3	20	7.1	45	5.5	23	0.152
Uncertainty of measurement	10%	15%	20%	20%	11%	25%	15%	10%	-	-	-

^a^ anthracene, acenaphtene, acenaphthylene, phenanthrene, fluorene, fluoranthene, benzo(a)anthracene, chrysene, pyrene, triphenylene, benzo(a)pyrene, benzo(b)fluoranthene, benzo(e)pyrene, benzo(k)fluorathene, dibenzo(a,h) anthracene, perylene, benzo(ghi)perylene, indeno(1,2,3-cd)pyrene, naftalene, 1-methylnaftalene, 2-methylnaftalene

### Nucleic acid extraction and cDNA synthesis

DNA from water samples was extracted using the NucleoSpin Soil DNA extraction kit (Macherey-Nagel, Düren, Germany). The frozen CA filters were thawed on ice and cut into two pieces each. Each membrane half was inserted in a bead tube for microbial cell lysis. The DNA was extracted using lysis buffer SL1 with Enhancer solution SX according to the manufacturer’s instructions. After the lysis step the supernatant of the filter pairs halves were combined for the rest of the extraction protocol. The DNA was eluted into 100 μl elution buffer SE.

RNA was extracted from the Sterivex filtration units using the NucleoSpin RNAPlant kit (Macherey-Nagel). The filter units were briefly thawed on ice after which they were cut open using sterile pliers and scalpels in a laminar flow hood. The filters were inserted into sterile 5-mL Eppendorf tubes equipped with approximately 2 mL of 150–212 μm diameter acid washed glass beads (Sigma-Aldrich, Munich, Germany) that had been heat sterilized at 250°C for 18 h before use. The RNA extraction proceeded with the RA1 buffer and ß-mercaptho ethanol according to the manufacturer’s instructions, with the exception of using 3-fold buffer volumes and lysing the microbial cells by vortexing horizontally at full speed for 5 min. The lysate was removed for further treatment according to the manufacturer’s instructions and the glass beads were rinsed with an additional 100 μl of RA1 and ß-mercaptho ethanol solution, which was added to the lysate. The RNA was finally eluted in 45 μl (180 mL samples) or 60 μl (240 mL samples) RNase-free water.

The frozen sediment tubes were thawed on ice and mixed by shaking in order to homogenize the sediment for DNA extraction. Triplicate 1 g subsamples of sediment were suspended in 2.5 mL buffer (10 mM Tris-HCl pH 7.5, 15 mM CaCl_2_, 60 mM MgCl_2_) and thoroughly mixed by vortexing. Three replicate 350 μl sediment slurry samples (equal to 100 mg sediment) were set aside for direct DNA extraction and the same amounts of subsamples were treated with DNase I in order to remove extracellular soluble DNA. To each 350 μl slurry sample 40 μl DNase I (4229 U mL^-1^, Thermo Fisher Scientific) was added. The reactions were incubated at 37°C for 3 h with occasional mixing by hand. The DNase reaction was terminated by heating at 65°C for 10 min. Excess supernatant, with possible trace amounts of external DNA, was removed from the DNase treated samples by centrifugation in a table top centrifuge (Eppendorf, Hamburg, Germany) at 11,000 g for 2 min where after the supernatant was decanted. The sediment suspensions that were not DNase treated were continued with as such. DNA was extracted with the NucleoSpin Soil DNA extraction kit according to the manufacturer’s instructions, using lysis buffer SL1 and enhancer solution. The concentration of DNA and RNA was measured with the Qubit 2.0 Fluorometer (Invitrogen, Life Technologies, CA, USA) according to the manufacturer’s instructions.

The extracted RNA was checked for residual DNA by end point PCR using primers U968f and U1401r [21] targeting the bacterial 16S rRNA gene. A PCR product was visible in most reactions after agarose gel electrophoresis and additional DNA removal was applied. Parallel DNase reactions were prepared for each sample. The reactions consisted of 13.8 μl RNA extract, 1.7 μl 10x reaction buffer and 1.7 μl DNase RQ1 (1000 U mL^-1^, Promega, WI, USA) and were incubated at 37°C for 30 min, after which the DNase reaction was inactivated by adding 1.8 μl DNase Stop Solution followed by a final 10 min incubation at 65°C. The parallel reactions were combined for reverse transcription (RT) of RNA to cDNA. The RT-PCR was conducted using the SuperScript III reverse transcriptase (Thermo Fisher Scientific) in two parallel reactions for each sample. The reaction proceeded in two steps. In the first step, 17.2 μl DNase treated RNA, 0.375 μg random hexamers (Promega) and 15 nmol dNTP (Promega) were mixed and incubated at 65°C for 5 min and cooled on ice. To the cooled reaction mixture, 6 μl 5x First strand buffer, 60 u DTT, 60 u Recombinant RNase inhibitor (Promega) and 300 u of Superscript III reverse transcriptase were added. The reactions were mixed by carefully pipetting the mixture, followed by a 5 min incubation at 25°C and a 30–60 min incubation at 50°C. The reaction was inactivated by heating at 70°C for 15 min. The cDNA was stored at -80°C until use.

### Estimation of microbial community sizes using qPCR and flow cytometry

The bacterial, archaeal and fungal biomass in the seawater and sediment was evaluated from the DNA extracts of the water samples and the sediment and from the DNase treated sediment with quantitative PCR (qPCR) using the LightCycler 480 instrument (Roche Diagnostics, Basel, Switzerland). For Bacteria, an approximately 200-bp fragment of the 16S rRNA gene was amplified with primers P1 and P2 [22]. For Archaea, an approximately 400-bp fragment of the 16S rRNA gene was amplified with primers A344F [23] and A744R, modified from [24]. The amplification was done in a 10-μL reaction volume with KAPA Sybr Fast qPCR Master mix optimized for Roche LightCycler 480 (KAPA Biosystems, Wilmington, MA, USA), 150 nM of each primer for the bacterial 16S rRNA gene and 300 nM of each primer for the archaeal 16S rRNA gene, and 1 μL of sample DNA. The amplification reaction consisted of initial denaturation at 95°C for 15 min, 45 cycles with 10 s at 95°C, 35 s at 57°C and 30 s at 72°C, a final elongation of 3 min at 72°C and a melting curve analysis.

Fungal biomass was detected with a TaqMan probe assay using the 5.8S rRNA gene as target for primers 5.8F1 (5′-AAC TTT CAA CAA CGG ATC TCT TGG-3′) and 5.8R1 (5′-GCG TTC AAA GAC TCG ATG ATT CAC-3′) and probe 5.8P1 (5'-CAT CGA TGA AGA ACG CAG CGA AAT GC-3’) [25]. The amplification was done in 10 μL reaction volume using the KAPA PROBE FAST qPCR Kit (KAPA Biosystems), 500 nM of each primer, 200 nM of probe, and 1 μL of template DNA. The amplification reaction consisted of enzyme activation at 95°C for 3 min and 40 cycles of 10 s at 95°C, 30 s at 62°C and 10 s at 72°C.

All qPCR reactions were conducted from triplicate DNA extractions with duplicate or triplicate reactions. Negative controls without added DNA template were included in each run. The number of bacterial and archaeal 16S rRNA and fungal 5.8S rRNA gene copies were compared to the amplification results of standard DNA dilution series. For Bacteria, a plasmid dilution series containing the 16S rRNA gene of *E*. *coli* ATCC 31608 reaching concentrations from 10^2^ to 10^8^ copies per reaction was used. The archaeal amplification was compared to that of a dilution series of *Halobacterium salinarum* VTT E-103154^T^ genomic DNA using a 10-fold dilution series equal to 10^2^ to10^7^ cells per reaction. The fungal 5.8S rRNA gene numbers were compared to a dilution series of genomic DNA of *Aspergillus versicolor* VTT D-96667 spores with 10 to10^5^ spores per reaction. Inhibition of the amplification was tested by adding 10^7^ plasmid copies containing fragments of the *E*. *coli* 16S rRNA gene to reactions containing the same amount of DNA extract as used in qPCR reactions. The qPCR was performed with the 16S rRNA gene specific primers and the percentage of inhibition was calculated by dividing the Cp values of original samples with added plasmid copies by the Cp value of sample with only plasmid copies and multiplying the result with 100. No inhibition was detected in any of the samples as from all samples and replicates with added plasmid copies at least 100% of the addition was returned.

Bacterial cells and virus-like particles (VLPs) were measured from seawater samples by flow cytometry done according to [26], except that paraformaldehyde 1% (v/v) in phosphate-buffered saline, was used as a fixative. Three replicants were measured from each site. The cells were stained with SYBR Green I (Sigma-Aldrich Inc, St Louis, MO, USA) at a final dilution of 1:10 000 for 15 min in the dark in room temperature. MilliQ water was used to measure the background. Fluoresbrite 0.5-μm microspheres (Polysciences Inc., Warrington, PA, USA) were used as a size standard. Enumeration of the diluted samples (dilution factor 10; molecular biology grade TE buffer, AppliChem GmbH, Darmstadt, Germany) was carried out, using a CyFlow Cube 8 (Partec GmbH, Munster, Germany) flow cytometer. A sample of 25 μl (defined by the flow cytometry electrodes) was analysed, using a flow rate of 12 μl min^−1^. The data were acquired on a dot plot displaying green fluorescence (488 nm) versus side scatter signal, both on a logarithmic scale. The detection trigger was depicted as a green fluorescence. Data was analysed with FCS Express software. Enumeration of sediment bacteria by Epifluorescence Microscopy was done with two replicants from each site as described in [27].

### Quantification of oil degradation genes PAH-RHDα and *alk*B by qPCR

Quantification of the PAH ring hydroxylating deoxygenase gene (PAH–RHDα) (Gram-positive and Gram-negative Bacteria separately) abundances were performed by qPCR using the primers: Gram-positive (GP) forward 5’-CGG CGC CGA CAA YTT YGT NGG-3’ and reverse 5´-GGG GAA CAC GGT GCC RTG DAT RAA-3’ and Gram-negative (GN) forward 5’-GAG ATG CAT ACC ACG TKG GTT GGA-3’ and reverse 5’-AGC TGT TGT TCG GGA AGA YWG TGC MGT T-3’ [16], 2.5 pmol each. The 10 μL reaction contained also 2x SensiFAST SYBR No.ROX Mix (Bioline, London, U.K) 5 μL and 1 μL of template DNA. No-template controls were included in every run and all qPCR reactions were conducted from triplicate DNA extractions with duplicate reactions. To quantify the *alk*B gene copy numbers, coding for a rubredoxin-dependent alkane monooxygenase, primers [28] *alkB-f* (5´-AAY ACI GCI CAY GAR CTI GGI CAY AA-3´) and *alkB-r* (5´-GCR TGR TGR TCI GAR TGI CGY TG-3´) were used. Thermal cycling conditions were 3 min at 95°C, 45 cycles of 15 s at 95°C, 30 s at 54°C, 30 s at 72°C, and 10 s at 86°C for GP reaction and 83°C for GN reaction and a final elongation of 3 min at 72°C. For *alk*B the thermal cycling conditions were 3 min at 95°C, a touchdown amplification of 5 cycles of 15 s at 95°C, 30 s at 62°C (stepwise reduction to 58°C), 30 s at 72°C, and 10 s at 78°C followed by amplification of 40 cycles of 10 s at 95°C, 30 s at 57°C, 30 s at 72°C, and 10 s at 78°C followed by final elongation 3 min at 72°C. Melting curve analysis was performed after each run. Standards used in the analysis were *Escherichia coli* plasmids with PAH–RHDα inserts amplified and cloned from *Pseudomonas putida* G7 (GN) or *Mycobacterium vanbaalenii* DSM 7251 (GP) and from *Pseudomonas putida* (*alk*B). Standards from 10^1^ to 10^8^ gene copies μL^-1^ were used for the calibration curve. The average gene copy numbers were calculated per gram dry weight (DW) of sediment.

### Amplicon sequencing, analysis and annotation

The amplification libraries for high throughput sequencing on the Ion Torrent PGM platform were prepared by PCR from the DNA and cDNA samples. Bacterial 16S genes were amplified with primers S-D-Bact-0341-b-S-17/S-D-Bact-0785-a-A-21 [29], targeting the variable region V3-V4 of the 16S rDNA gene, archaeal 16S rDNA genes with primers S-D-Arch-0349-a-S-17/S-D-Arch-0787-a-A-20 [30], targeting the V4 region of the gene, and fungal internal transcribed spacer (ITS) gene markers with primer pair ITS1 and ITS2 targeting the fungal ITS1 region [31, 32].

PCR amplification was performed in parallel reactions for every sample containing 1× MyTaqTM Red Mix (Bioline), 20 pmol of each primer, 2 μL of template and nuclease-free water (Sigma-Aldrich) to a final reaction volume of 25 μL. The PCR program consisted of an initial denaturation step at 95°C for 3 min, 35 cycles for Bacteria and Fungi and 40 cycles for Archaea of 15 s at 95°C, 15 s at 50°C and 15 s at 72°C. A final elongation step of 30 s was performed at 72°C. Correct size of the PCR products was verified with agarose gel electrophoresis. Parallel amplicon libraries were combined and sequenced by Bioser, University of Oulu (Finland) on an Ion Torrent PGM sequencer (Thermo Fisher Scientific) using the 314 and 316 Chip Kit v2 with the Ion PGM Template IA 500 and Ion PGM Hi-Q sequencing kits. The amplicons were filtered for correct size (200–600 bp) and purified prior to sequencing.

The sequence reads obtained from Ion Torrent sequencing were subjected to quality control using the QIIME-software Version 1.9 [33] using a minimum quality score of 20, minimum and maximum sequence length of 200 bp and 600 bp, respectively, maximum primer mismatch of 2 nucleotides (nt) and maximum homopolymer stretches of 8 nt. Adapters, barcodes and primers were removed from the sequence reads, and chimeric sequence reads were removed from the dataset with the USEARCH-algorithm [34] by *de novo* detection and through similarity searches against the Silva seed version 128 database [35] with bacterial and archaeal sequences or the UNITE database (version 7.1) [36] for fungal ITS sequences. Bacteria and archaeal OTUs were picked at 97% sequence homology against the Silva database using the *de novo* OTU picking strategy in QIIME. Fungal ITS OTUs were picked at dynamic 97 to 100% sequence homology against the UNITE database using the *de novo* OTU picking strategy in QIIME. Taxonomic assignments for all sequences were made using the BLAST algorithm with a maximum E-value of 0.001 [37]. Singleton, unassigned and unidentified OTUs were filtered from the datasets.

The sequences were deposited in the European Nucleotide Archive (ENA, https://www.ebi.ac.uk/) under accession number PRJEB31590.

### Statistical analyses

Alpha-diversity measures (observed OTUs, Chao1 richness estimate and Shannon’s diversity index) were calculated based on the raw OTU data outputted by QIIME using the Phyloseq package in R [38, 39] and visualized using ggplot2. Significant differences in the number of OTUs, the Chao1 richness estimates and Shannon indices were tested with two sample paired t test in PAST 3.14 [40]. Correlation (Pearson’s r) between qPCR copy numbers, DAPI and flowcytometry cell counts were also tested in PAST 3.14.

The similarity of the archaeal, bacterial and fungal communities between the different sample sites was tested by principal coordinate analysis (PCoA) using the Phyloseq package in R. The analysis was performed using the relative abundance OTU tables outputted by QIIME. The Bray-Curtis distance model was used for both analyses. Eigen values for the variance explained by the PCoA dimensions were calculated on 999 random repeats. Statistically significant (p<0.05) environmental parameters were added to the ordinations with the Vegan package in R with 999 permutations for water and sediment samples separately. Significant differences in the relative abundances of genera of the different sample sites were compared in QIIME (group_significance.py) with one way analysis of variance (One-way ANOVA) and Bonferroni corrected p-values.

## Results

### Chemical composition of water and sediment

The two studied coastal sites were around 200 km apart from each other and had only minor differences in the water and sediment characteristics (Tables 2 & 3). The water salinity ranged from 5 to 5.7 PSU in the sample sites, being slightly higher in the Naantali site [19, 20]. The salinity tends to increase as a function of depth, and in the Porvoo site the increase was around 0.3 to 0.6 PSU and in Naantali only 0.1 PSU higher at the sea floor. In general, the chloride, sulphate, total nitrogen, nitrate, nitrite, total phosphorus, phosphate phosphorus and total iron concentrations were at the same level in both study site waters as were also the organic matter and elemental N, C and S levels in the sediments. However, PAH concentrations were much higher in the Porvoo sediment samples D and Q, compared to the other sampling points. Likewise, the petroleum hydrocarbon concentration was high in these two samples, especially in Porvoo D sediment sample, compared to other sediment samples. PAH concentrations were higher in water samples taken from Naantali than in Porvoo samples whereas the petroleum hydrocarbon concentrations were higher in Porvoo water samples.

**Table 3:** Elemental composition, petroleum hydrocarbons and total PAH concentrations in sediment samples.

Site	Organic matter % dw	N % dw	C % dw	S % dw	C_10_-C_40_ mg kg^-1^ dw	Total PAHs ^a^ μg kg^-1^ dw
Porvoo Q	7.4	0.3	3	0.1	150	2317
Porvoo D	11.5	0.2	2.1	0	1085	41305
Porvoo B	11.2	0.4	5.6	0.2	204	524
Naantali PP	5.5	0.6	4.6	0.6	165	554
Naantali 300	7.7	0.4	2.7	0.2	38	326

^a^ anthracene, acenaphtene, phenanthrene, fluorene, fluoranthene, benzo(a)anthracene, chrysene, pyrene, triphenylene, benzo(a)pyrene, benzo(b)fluoranthene, benzo(e)pyrene, benzo(k)fluorathene, dibenzo(a,h) anthracene, perylene, benzo(ghi)perylene, indeno(1,2,3-cd)pyrene, naftalene, 1-methylnaftalene, 2-methylnaftalene

### Community sizes (Dapi, Flow, qPCR)

**Fig 2:**
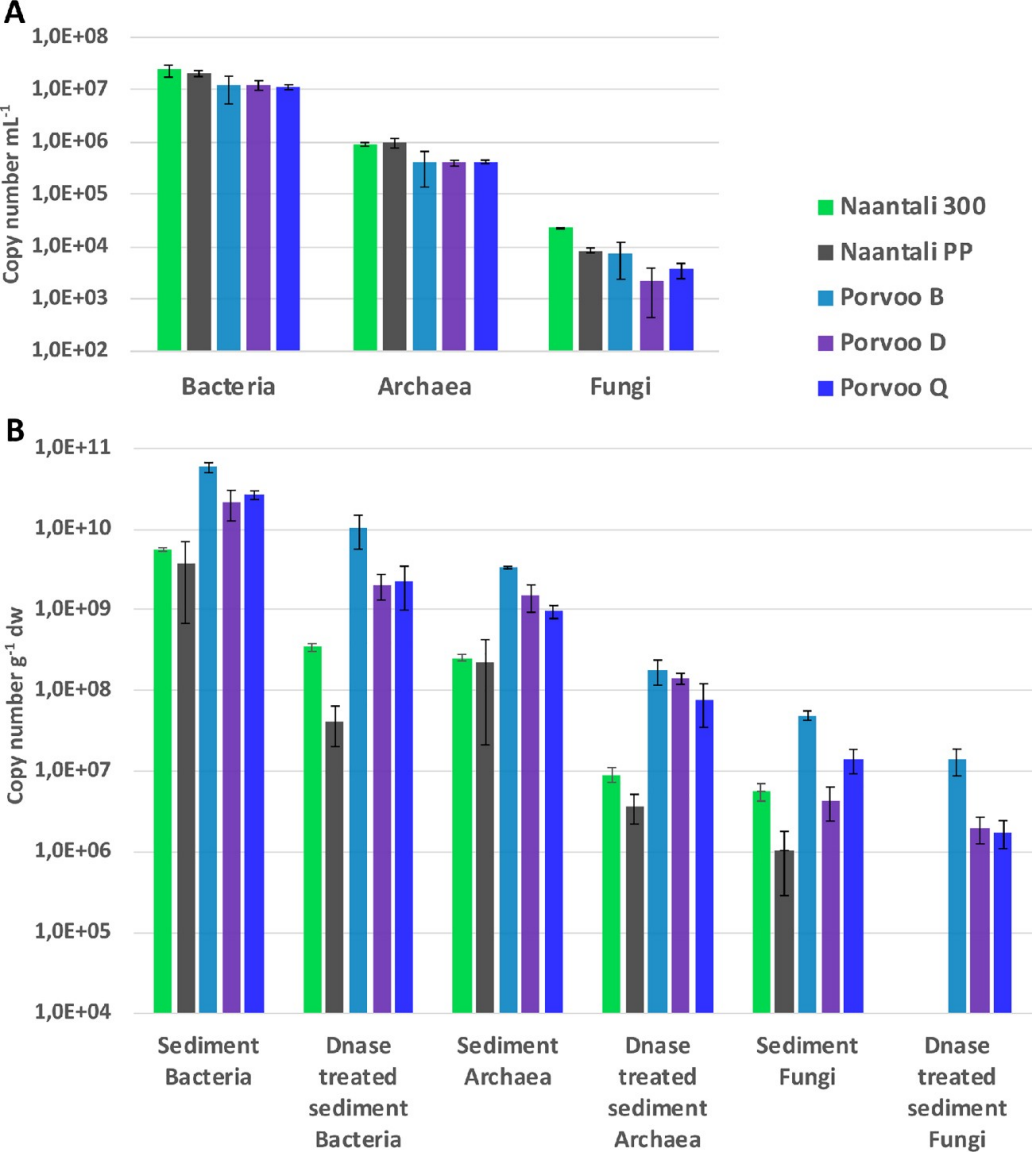
Number of bacterial and archaeal 16S rRNA and fungal 5.8S rRNA gene copies. Copy numbers determined by qPCR from triplicate DNA extractions from (A) sea water (mL^-1^) and (B) untreated sediment or sediment treated with DNase (g^-1^ dry weight).

The number of cells in the water samples was on average 6.0 x 10^6^ mL^-1^ based on flow cytometry (Table 4). The average number of virus-like particles (VPL) was 2.8 x 10^7^. The number of cells in the sediment samples was on average 9.6 x 10^5^ g^-1^ dry weight, analysed with epifluorescence microscopy (Table 4). The variation in cell numbers between the sites was small in the water samples, only 0.2 logarithmic units (Table 4). However, there was statistically significant (p<0.01) negative correlation (r = -0.97) between the cell counts and the petroleum hydrocarbon (C_10_-C_40_) concentrations in water, whereas PAH-compounds had an opposite effect on the cell count (p<0.01, r = 0.99). In the sediments, no statistical correlation was found between the number of cells and concentration of petroleum hydrocarbons or PAHs. However, in the reference sites located at a distance from the refineries (300 and B) the number of cells in the sediments were higher than in the samples from the sites located close to refineries (Table 4). The same trend was also visible in the number of bacterial and archaeal 16S rRNA and fungal 5.8S rRNA gene copy numbers analysed by qPCR from sediments. These were always lower in samples taken close to the refineries (PP, D and Q) than in their reference samples (300 and B, Fig 2). The qPCR results showed that the average number of bacterial 16S rRNA gene copies was over one logarithmic unit higher in general than the number of archaeal 16S rRNA gene copies in sediments (S1 and S2 Tables). The fungal 5.8S rRNA gene qPCR assay also showed that Fungi were abundant in all samples with the exception of the DNase treated sediment from Naantali.

**Table 4:** Bacterial cells in sediment (DAPI) and bacterial cells and virus-like particles (flow-cytometer) in water samples.

Site	Sediment		Water			
	Cells g^-1^ dw	SD	Cells mL^-1^	SD	VLPs ml^-1^	SD
Porvoo Q	4.8 × 10^5^	1.2 × 10^5^	6.7 × 10^6^	8.8 × 10^5^	2.1 × 10^7^	8.6 × 10^5^
Porvoo D	9.8 × 10^5^	4.5 × 10^5^	5.4 × 10^6^	7.2 × 10^5^	1.9 × 10^7^	1.2 × 10^6^
Porvoo B	1.9 × 10^6^	1.6 × 10^6^	5.7 × 10^6^	1.5 × 10^5^	2.0 × 10^7^	8.5 × 10^5^
Naantali PP	1.2 × 10^6^	5.4 × 10^5^	6.9 × 10^6^	6.1 × 10^5^	3.8 × 10^7^	4.3 × 10^6^
Naantali 300	2.3 × 10^5^	4.4 × 10^4^	5.2 × 10^6^	1.4 × 10^6^	4.1 × 10^7^	3.9 × 10^6^

**Fig 3:**
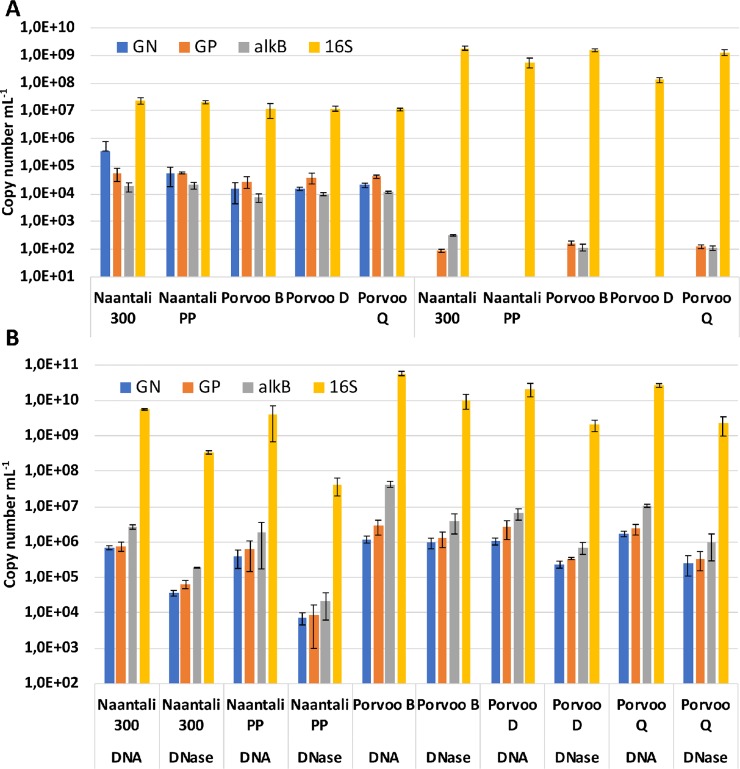
Number of PAH-RHDα Gram-negative (GN), Gram-positive (GP), *alk*B and bacterial 16S rRNA genes copies. Copy numbers determined by qPCR from triplicate DNA and RNA sample extractions isolated from A) sea water (mL^-1^) B) untreated sediment or sediment treated with DNase (g^-1^ dry weight).

The number of oil degradation genes *alk*B, PAH-RHDα Gram-positive (GP) and PAH-RHDα Gram-negative (GN) copy numbers in water samples were mostly between 10^4^ to 10^5^ mL^-1^ (Fig 3A, S3 Table). There was statistically significant (p<0.05) correlation between *alk*B and PAH-RHDα GP gene copy numbers with bacterial and archaeal 16S rRNA gene copy numbers, PAH-compound concentration (r>0.88) and negative correlation with petroleum hydrocarbon concentration (r<-0.92). The number of transcripts from the RNA extractions of the water samples was 10^2^ mL^-1^ or at the detection limit for *alk*B and PAH-RHDα GP genes, respectively, but below the detection limit for PAH-RHDα GN gene (Fig 3A).

In sediments and in DNase treated sediments the oil degradation gene copy numbers ranged from 10^4^ to 10^7^ g^-1^ dry weight (Fig 3B, S4 Table). The number of oil degradation genes in sediment samples showed statistically significant (p<0.015) correlation with the copy numbers of bacterial 16S rRNA gene (r = 0.74 to 0.96) and the location near refinery seemed not to affect the oil degradation gene abundance. Additionally, the PAH compound and petroleum hydrocarbon concentrations did not correlate with the oil degradation gene copy numbers. 16S rRNA gene copy numbers were three to four and two to three logarithmic units higher than the oil degradation gene copy numbers in sediments and in water, respectively.

The DNase treatment method used before DNA extraction was targeted to remove the extracellular soluble DNA fraction from the sediment samples. The treatment removed from 100% to 83.7% of the DNA in the triplicate sediment samples compared to DNA extractions without the DNase treatment (S5 Table). Especially in the Naantali samples the DNase treatment resulted in differences in both fungal and archaeal communities based on qPCR and sequencing.

### Microbial community composition by amplicon sequencing

In general, the number of OTUs found in the RNA fraction of the water samples was statistically significantly higher (p<0.01) than that detected in the DNA fraction of the same samples in all studied domains of Bacteria (in average 2304/1453 OTUs from RNA/DNA fractions), Archaea (2770/1150) and Fungi (1038/858). Shannon’s diversity index (*H’*) for each domain from the RNA fraction of the water samples was also statistically significantly higher (p<0.02) than for DNA (S2 Table). DNA samples may have been influenced by the longer transportation and storage at +4°C times (12-48h) before filtrating the samples compared to the RNA samples (3–8 h storage). In sediment samples the OTU richness for Bacteria (12150/7023), Archaea (4860/814) and for Fungi (1840/1019) were statistically significantly (p<0.02) higher for untreated sediment samples than for DNase treated sediments, respectively. In addition, the diversity indices were also statistically significantly higher (p<0.01, one parallel outlier removed) for the untreated sediment samples than for the DNase treated sediment samples in the all studied domains. This seems logical as the DNase treatment was expected to remove the extracellular soluble DNA in the samples and also reduce the numbers of detected OTUs. Based on the sampling locations, the RNA and DNA fractions of the water had statistically significantly (p<0.01) more OTUs and the OTU richness was statistically significantly higher (p<0.01) in Naantali than in Porvoo water samples. Nevertheless, there were no statistically significant differences in the diversity indices between the two locations.

**Fig 4:**
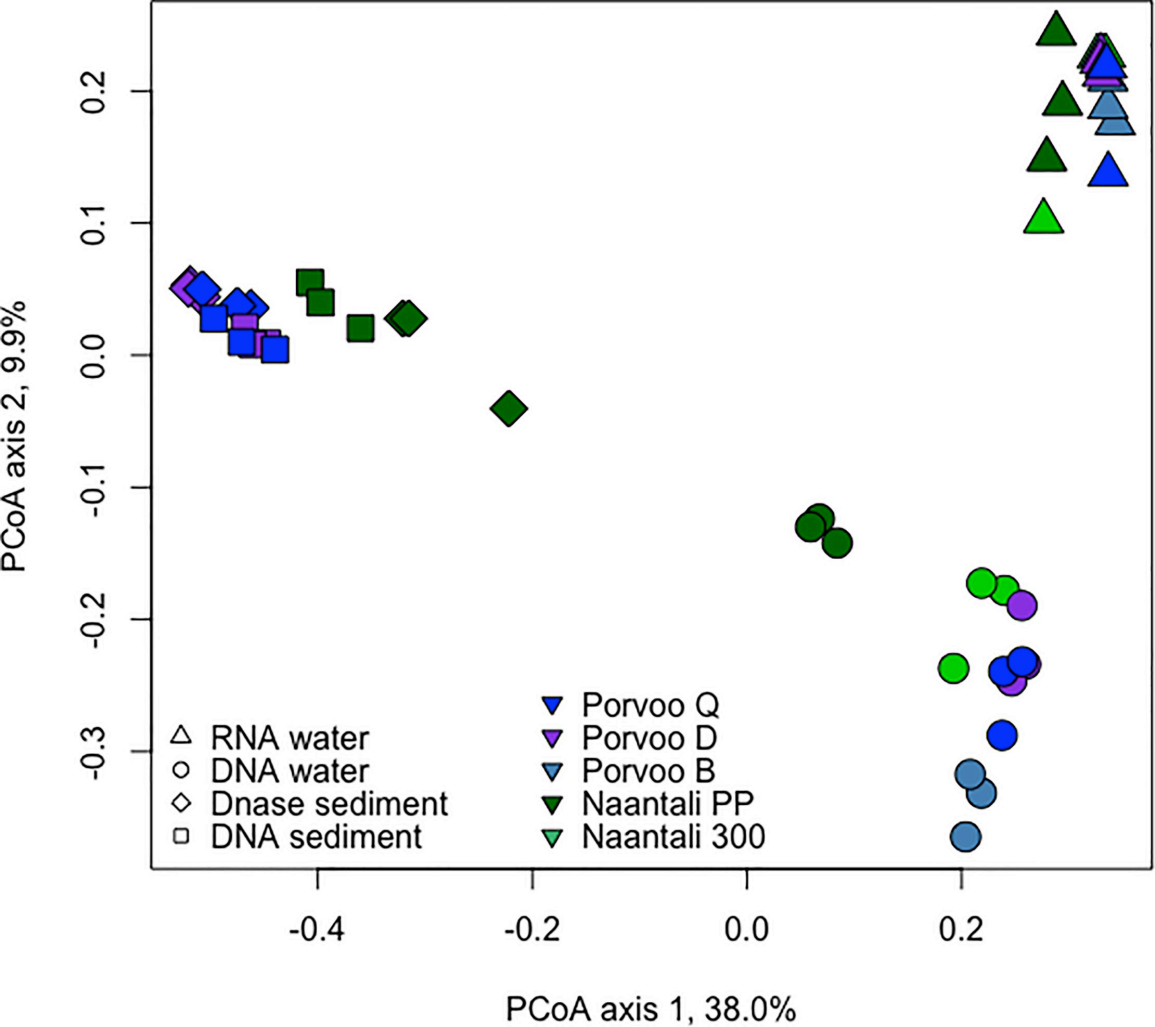
Principal coordinates analysis of combined bacterial, archaeal and fungal community compositions. PCoA from water and sediment samples. No archaeal sequence data was obtained from Naantali 300 and Porvoo B sediment samples, and these samples are removed from the analysis.

**Fig 5:**
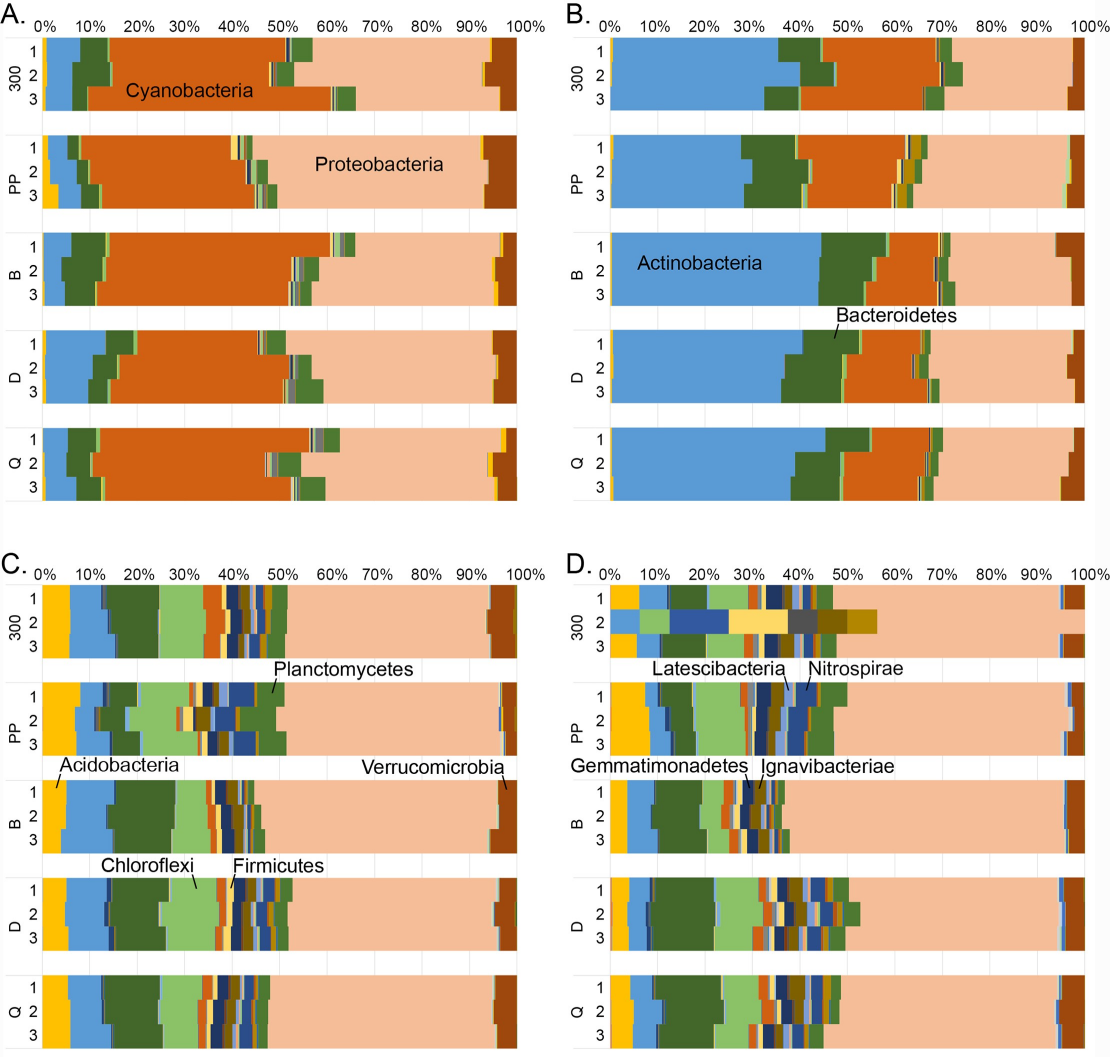
Relative abundance of bacterial phyla. Bacteria detected from A) RNA, B) DNA isolated from the sea water samples, C) DNA isolated from the DNase treated sediment samples and D) DNA extracted directly from the sediment samples.

Differences in the community structures in the studied samples were tested by Principal Coordinates analysis (PCoA). Water and sediment samples were clearly separated from each other on the PCoA plots (Fig 4). Similarly, based on analysis that combined the bacterial, archaeal, and fungal diversity, DNA fractions from water samples were clearly separated from RNA fractions (Fig 4). When analysing only the Bacteria, the Naantali and Porvoo DNA fractions grouped in close vicinity of each other (S1 Fig). The bacterial communities identified from DNA and RNA samples isolated from water samples consisted mainly of the same 46/47 bacterial phyla, however the distributions of these phyla were different (Fig 5). In RNA samples the share of dominating Cyanobacteria and Proteobacteria sequences were higher (25–51% and 22–48%) than in DNA samples (10–26% and 23–31%). In the DNA samples the share of Actinobacteria (28–45%) and Bacteroidetes (7–14%) was higher than in the RNA samples (4–13% and 2–10%, respectively). The Proteobacteria detected in water samples were mainly Alphaproteobacteria, the share being higher in RNA fraction (16–26%) than in DNA fraction (14–17%). Less than 10% of all bacterial sequences were identified as Beta- or Gammaproteobacteria. The amount of Deltaproteobacteria was only 1–2% in DNA-water samples but slightly higher in RNA-water samples (2–6%). In addition, Planctomycetes (1–6%) and Verrucomicrobia (2–7%) were detected in all water samples.

In untreated and DNase treated sediment samples, the number of detected bacterial phyla was higher than in the water samples, i.e. 62 and 56 phyla, respectively. The bacterial diversity in the sediment samples differed significantly from the water samples, but the two sampling locations did not differ markedly from each other (Fig 4). The share of Cyanobacteria and Actinobacteria sequences was lower whereas the share of Acidobacteria, Chloroflexi and Proteobacteria sequences was higher compared to the water samples (Fig 5). In contrast to the bacterial communities in the water samples, the Proteobacteria consisted mainly of Gammaproteobacteria (14–29%). Alphaproteobacteria that were abundant in water samples were found only at around 4% (2–6%) of the bacterial sequences in the sediment samples which was also the share of the Betaproteobacteria (2–8%). The share of Deltaproteobacteria was significantly higher in sediment (10–22%) than in water samples. In addition, some Epsilonproteobacteria were also found in sediment samples (1–2%). From all sediment samples, several other phyla were found with approximately 1% to 5% relative abundances: Verrucomicrobia, Planctomycetes, Nitrospirae, Ignavibacteriae, Gemmatimonadetes, Latescibacteria, Firmicutes and Parcubacteria.

**Fig 6:**
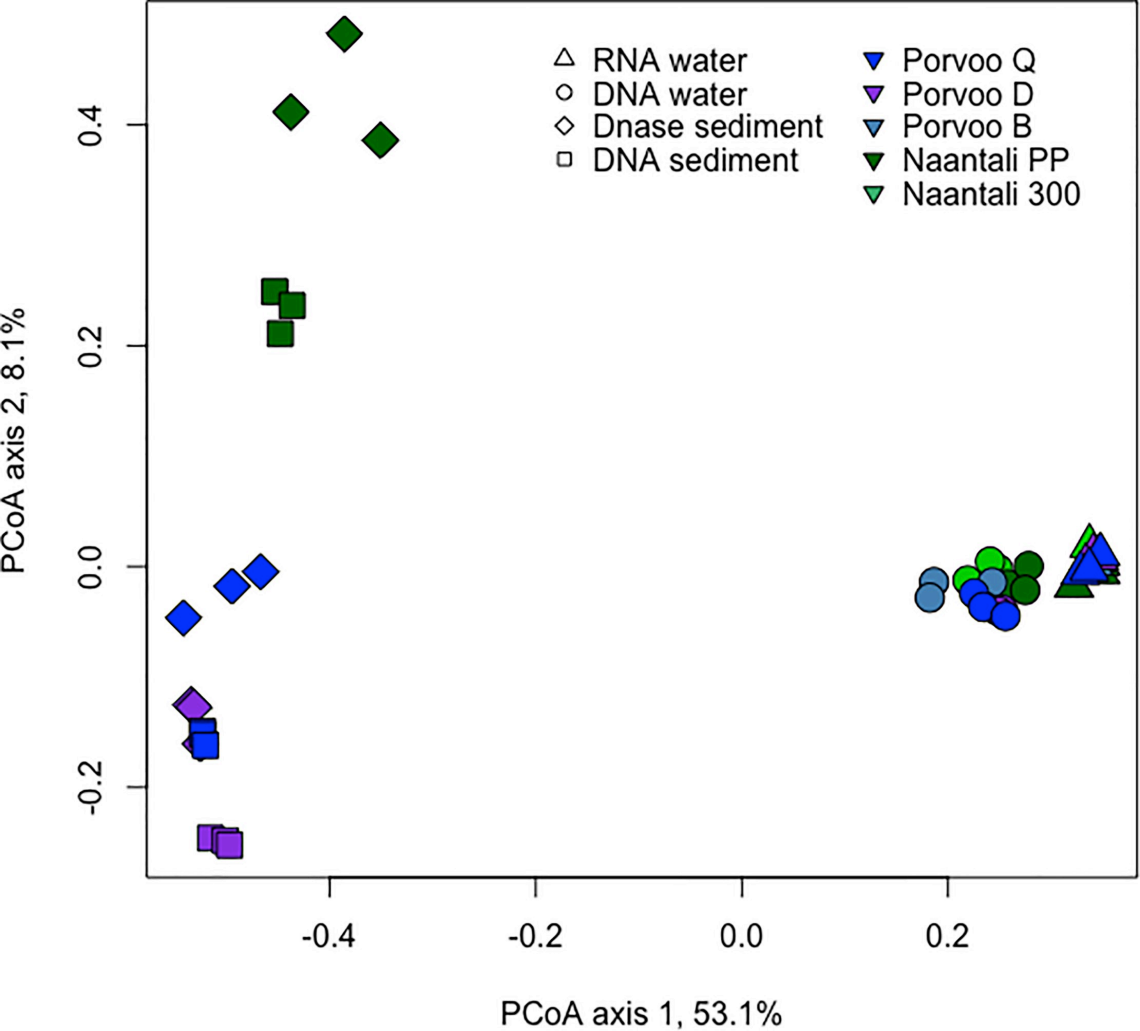
Principal coordinates analysis of archaeal sequences from water and sediment samples. No sequence data was obtained from Naantali 300 and Porvoo B sediment samples.

**Fig 7:**
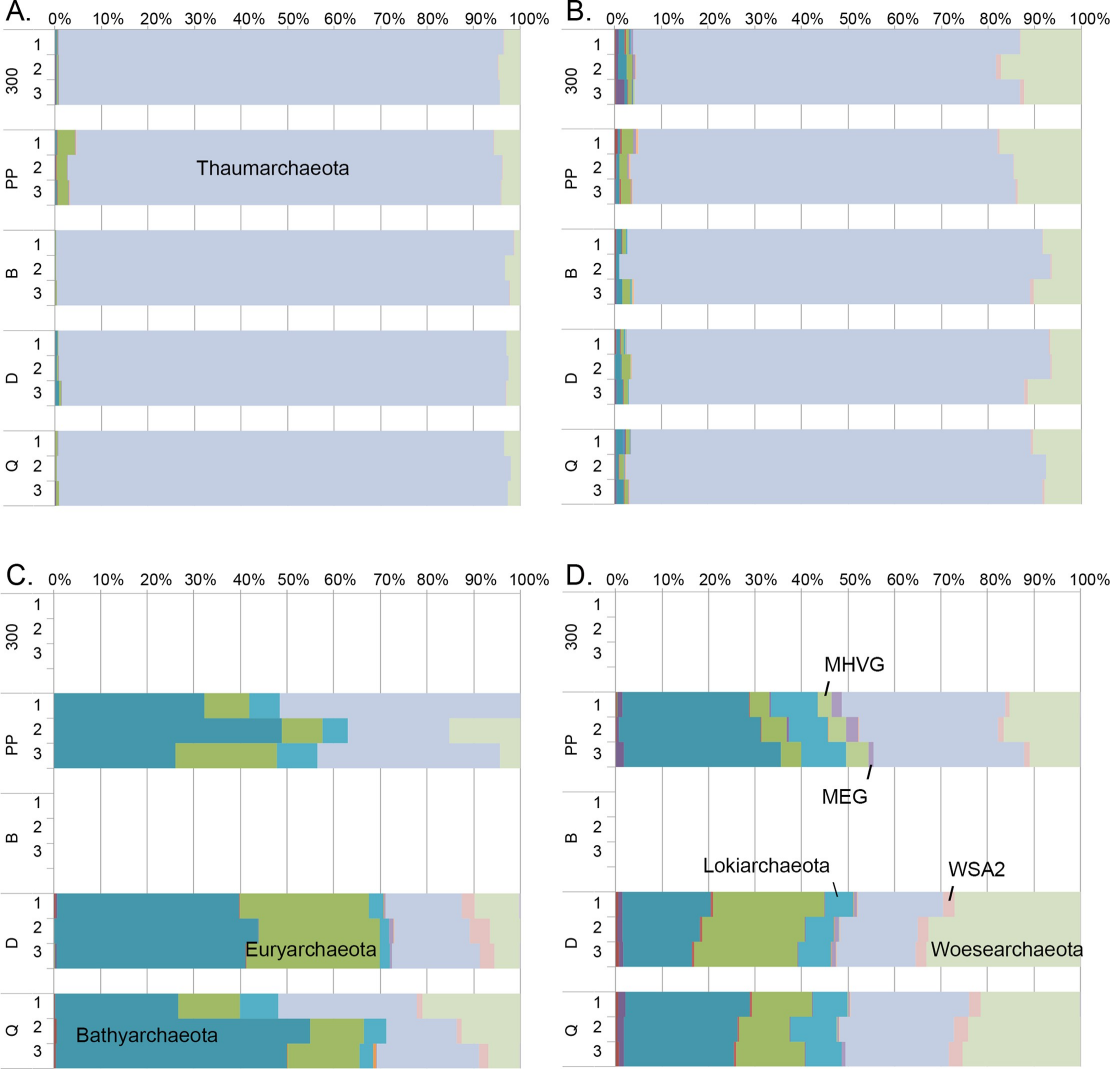
Relative abundance of archaeal phyla. Archaea detected from A) RNA, B) DNA isolated from the sea water samples and C) DNA isolated from the DNase treated sediment samples and D) the DNA extracted directly from the sediment samples.

In the PCoA of the archaeal communities, water and sediment samples were again clearly separated from each other (Fig 6). There was a small visible difference in water DNA and RNA samples ordination regardless of their sampling site. The number of archaeal phyla detected was 18 in both RNA and DNA water samples. Thaumarchaeota dominated both in the RNA (93–98%) and DNA (77–92%) fractions of the water samples (Fig 7). The rest of the sequences were mainly identified as Woesearchaeota that were more abundantly detected from the DNA fraction of the water samples (6–18%) than in RNA fractions (1–6%). Bathyarchaeota and Euryarchaeota were also frequently detected at low relative abundance. The archaeal diversity was higher in sediment samples than in water samples. Nineteen archaeal phyla were detected in the DNase treated sediment and 23 in the untreated sediment samples. No sequences were obtained from the two reference locations (B & 300) sediments. The archaeal community profiles of DNase treated, and untreated sediments were quite similar, but the average Shannon diversity index was three units higher (8.8/5.8 units) in untreated sediments which was a far greater difference than observed in the bacterial and fungal results (<1 unit) from the same sediments (S6 Table). According to PCoA (Fig 6) sediment samples grouped based on the sampling location (Naantali/Porvoo) although the reference locations (B & 300) with very low numbers of archaeal sequences were removed from the analysis. The dominant phyla in the sediment samples were Bathyarchaeota (15–54%), Euryarchaeota (4–29%), Thaumarchaeota (15–52%) and Woesearchaeota (0–39%). In addition, Lokiarchaeota (2–10%), MHVG (0–5%) and WSA2 (0–4%) were found frequently (Fig 7).

**Fig 8:**
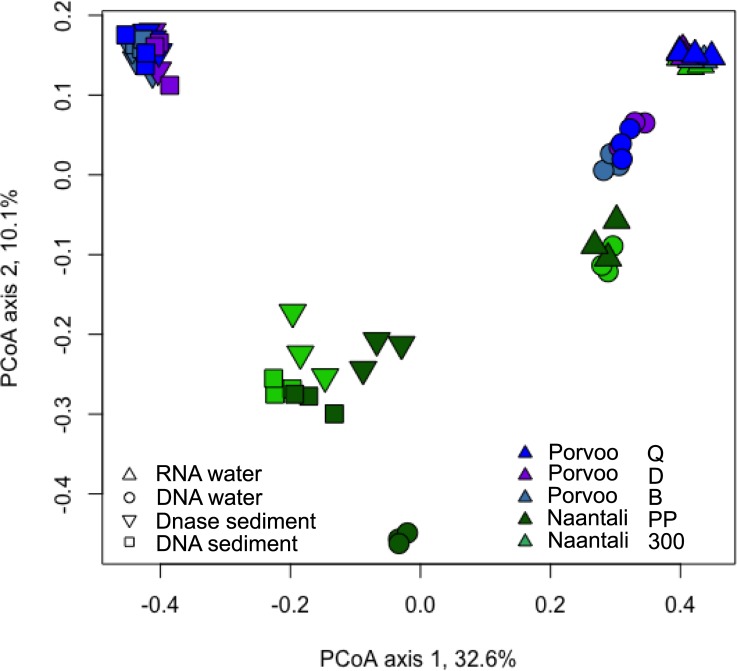
Principal coordinates analysis of fungal sequences from water and sediment samples.

**Fig 9:**
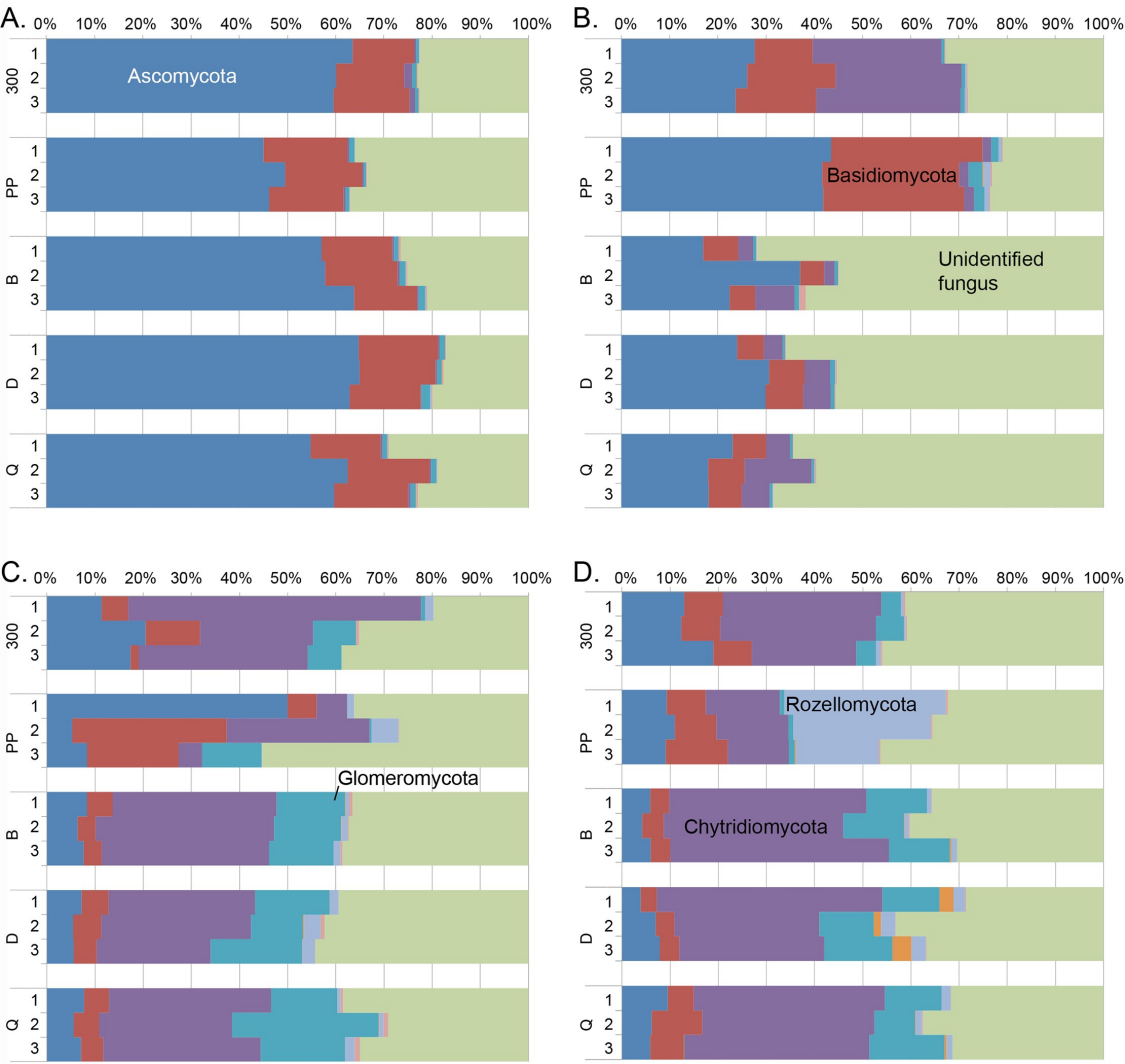
Relative abundance of fungal phyla.

Similar to the bacterial and archaeal analysis, PCoA of fungal sequences separated water and sediment samples. However, no clear grouping of water samples based on the DNA and RNA fraction or sampling site was visible (Fig 8). Similar to the archaeal sequences in sediment samples were, instead, grouped based on the sampling site. Altogether 9 fungal phyla were observed in water or sediment samples. RNA fractions isolated from the water samples were dominated by Ascomycota sequences (Fig 9). In addition, Basiodiomycota and an unidentified fungal clade were abundant. These same phyla were found in DNA fractions from water samples in addition to a high relative abundance of Chytridiomycota. Contrary to the bacterial and archaeal community profiles, the sampling location seemed to influence the fungal communities more than analysed nucleic acid fraction or DNase treatment (Fig 8). In DNA fraction isolated from water samples from the Naantali locations harboured more Basiodiomycota compared to Porvoo samples. The reference sample (300) had also abundantly Chytridiomycota (Fig 9). In DNA isolated from Porvoo DNA-water samples an unidentified fungus dominated instead. In sediment samples the share of Chytridiomycota and Glomeromycota was higher than in water samples, otherwise the same major phyla were found from sediments as from water samples. In addition, in Naantali PP sample a higher relative abundance of Rozellomycota was detected compared to other sample locations.

### Environmental parameters and oil degradation potential

Environmental parameters influenced only slightly the microbial community compositions of the water samples (S2 Fig) when bacterial, archaeal and fungal results were combined. Porvoo samples grouped sta. sign. towards Petroleum hydrocarbon (C_10_-C_40_), phosphate and sulphate concentrations whereas Naantali samples grouped sta. sign. towards salinity, total nitrogen, phosphorus and iron concentrations. The effect of environmental parameters was most prominent when only bacterial community composition was analysed (S3 Fig).

**Fig 10:**
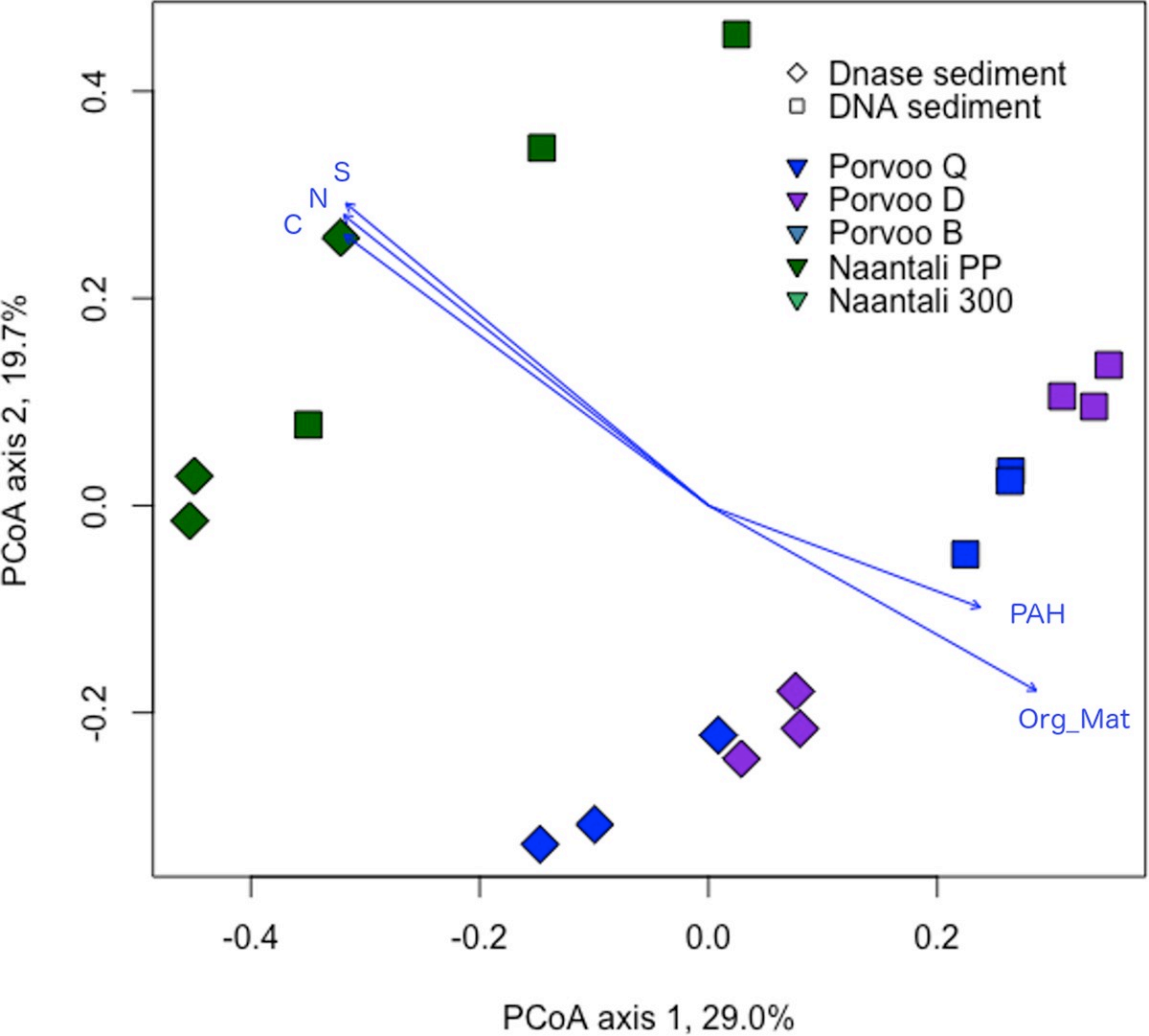
Principal coordinate analysis of combined bacterial, archaeal and fungal community composition and environmental parameters from sediment samples. All presented environmental variables were statistically significant (p<0.05) with 999 permutations. No archaeal sequence data was obtained from Naantali 300 and Porvoo B sediment samples, and these samples are removed from the analysis.

The microbial communities in the sediment samples were more affected by the measured environmental parameters. Samples collected from Porvoo grouped towards higher PAH and organic matter concentrations and Naantali samples towards higher nitrogen, carbon and sulphur concentrations (Fig 10). In addition to sampling location, the fungal sediment communities were separated based on environmental parameters (S4 Fig). Both PAH compounds and petroleum hydrocarbon concentration affected the fungal communities of Porvoo locations D and Q close to the refinery whereas Porvoo reference sample B was more affected by the organic matter. Naantali near refinery samples (PP) grouped towards high nitrogen and sulphur concentration in contrast to the Naantali reference sample (300).

In Porvoo sediment sample D the PAH concentration was 18 to 127 times higher than in the other sediment samples (Table 3). The fungal taxon Glomeraceae was detected in sediment D at statistically significantly (p<0.002) higher relative abundance than in other sediments. Another fungal taxon statistically significantly (p<0.02) more abundantly found in sediment D than in other sediments was Caloplaca, a lichen genus. Similar patterns were seen in the bacterial communities with statistically significantly (p<0.001) higher relative abundance of one Dehalococcoidia (Napoli-4B-65), one Acetothermia, three Bacteroidetes and one Anaerolineaceae taxon in sediment sample D compared to the other sediment samples. Archaeal taxa with statistically significantly (p<0.001–0.04) higher relative abundance in sediment D compared to the other sediment samples included Methanosaeta, several euryarchaeotal taxa including anaerobic methane oxidizers (ANME 2 and ANME 3), methanogenic Archaea (Methanomicrobiaceae, Methanosarcinaceae, ‘*Candidatus Methanofastidiosa’*) and Thermoplasmatales.

## Discussion

### Community sizes and functional genes involved in petroleum hydrocarbon degradation

The number of bacterial cells in sea water can vary depending on various factors such as location, organic matter present and temperature [41]. Furthermore, the amount and diversity may be affected by grazing by zooplankton or viral lysis in the marine ecosystem [42]. The amount of microorganisms in water and sediment samples were determined with traditional and molecular biology methods to compare the amounts with the numbers of oil degradation genes and environmental parameters. The number of cells in the water samples was in line with earlier reports of around 10^5^ to 10^7^ cells mL^-1^ in the Baltic Sea [43–45]. The number of virus-like particles was 10 times higher than the cell numbers in the water samples, indicating that viruses may also play a role in shaping the size of the community. The number of cells in the sediment samples was around 10^6^ cells g^-1^ DW in the pristine sites and 10^5^ in the polluted sites. In the study of Jankowska et al. [46] levels of 10^7^ cells g^-1^ DW were found in the Southern Baltic Sea sediments. However, the salinity and temperatures were lower and the depths greater in our study sites which may influence the community size. Llobet-Brossa et al. [47] reported on 10^9^ cells per cm^3^ of sediment in the Wadden Sea of the German North Sea coast, showing differences depending on sediment type and depth. Overall studies on the abundance of sediment bacteria can be difficult to compare because of different measurement units, sediment types, other environmental factors and methods used.

Based on both the water and sediment sample results the oil degradation gene copy numbers correlated with the number of bacterial/archaeal 16S rRNA gene copy numbers in the samples and not with the concentrations of petroleum hydrocarbons or PAH compounds. This indicates that the part of the community capable of or actively degrading oil compounds was probably relatively small compared to the total microbial community. However, the high gene copy numbers compared to the direct cell counts indicates that the microbes present includes a large fraction of taxa that have multiple copies of the 16S rRNA genes in their ribosomes. The *alk*B and PAH-RHD genes are often located on plasmids, which also seemed to be present in multiple copies in many organisms.

In three out of five water samples both the PAH-RHDα GP and *alk*B genes were transcribed at low levels in the water indicating possible PAH compound and alkane degradation. This is supported by measurable amounts of PAHs and petroleum hydrocarbons in the waters (Table 2). The copy numbers of PAH-RHDα GP genes in the total water community (DNA) were at the level of 10^4^ mL^-1^, similar to that of an earlier study of pristine and previously oil exposed waters from the Archipelago Sea of the Baltic Sea [48]. However, Reunamo et al. [48] detected PAH-RHDα GN genes only from the oil exposed water (10^2^ mL^-1^) whereas in the present study the PAH-RHDα GN gene copy numbers were higher than 10^4^ mL^-1^ in all water samples, showing relatively high presence of genes for oil degradation even though they were not actively transcribed. Alkane monooxygenase, *alk*B gene, was detected at the same level as the PAH-RHD genes, 10^4^ mL^-1^. The *alk*B gene has previously been detected in the Baltic Sea water and sediments but has not been quantified [49–51]. From the Gulf of Finland, the *alk*B gene was found from around 16% of the oil degrading bacterial strains, especially from the *Pseudomonas* and *Rhodococcus* genera [51]. These genera were both detected with low relative abundance in some of the samples in this study.

### Bacterial community compositions of sea water from RNA and DNA fractions

Previously, microbial communities in the surface waters of the Baltic Sea have been studied based on DNA only. Analyses of amplicon sequences of RNA and DNA fractions of the water samples in this study showed that, based on the abundance of the dominant phyla, the active (transcribed) bacterial, archaeal and fungal populations were different from the total communities. The difference was most noticeable in the bacterial communities where active communities in the water samples were dominated by Cyanobacteria and Proteobacteria whereas in total bacterial community Actinobacteria was the most abundant phylum in all studied sites. The Cyanobacteria and many Proteobacteria are known to respond quickly to suitable environmental conditions and may rapidly form blooms. Most of them are r-strategists and able to rapidly scavenge appearing nutrients in contrast to many Actinobacteria, which often are slower growing (k-strategists). Furthermore, Cyanobacteria are known to be host of many viruses [42], which may increase the viral loop, with lysis of the cells and rapid regrowth. This could possibly reflect the high transcription level of this group. Actinobacterial dominance based on DNA analysis is in line with earlier studies from the Baltic Sea during the summer season that show that the bacterial composition of water in several locations in the Baltic Sea area contain a high relative abundance of Actinobacteria together with Alphaproteobacteria, Bacteroidetes, Betaproteobacteria and Cyanobacteria and with lower relative abundance of Gammaproteobacteria, Verrucomicrobia and Firmicutes [52]. Herlemann et al. [29] also reported bacterial communities dominated by Actinobacteria, Alpha-, Beta- and Gammaproteobacteria as well as Cyanobacteria, Bacteroidetes and Verrucomicrobia in Baltic Sea locations with similar salinity and depth as in our water samples. However, Koskinen et al. [53] studied the bacterial diversity of three pelagic and one estuarine location in the North Baltic Sea in spring. Overall, they found a relatively low amount of bacterial classes, 23, of which Proteobacteria dominated (66%) with Bacteroidetes (13%) and Actinobacteria (4%) as the phyla with second and third highest relative abundances. This community composition is different from our results even though these phyla were detected frequently in our study.

### Bacterial community differences between water and sediment samples

The differences in Bacterial, Archaeal and Fungal communities between sediment and water samples were even more prominent than between active and total communities in water samples. This was seen in both sampling locations. Bacterial sequences affiliating with Acidobacteria, Chloroflexi and Proteobacteria were more abundant in the sediments than in the water samples. Possible low oxygen content and fluctuating nutrient conditions in the studied sediments may explain a large part of the occurring taxa in the sediment samples. Acidobacteria have the capacity to grow in different oxygen concentration gradients and to use diverse carbohydrates as well as organic and inorganic nitrogen sources [54]. Anaerolineae was the major class detected from the Chloroflexi phylum and the known Anaerolineae are anaerobic [55]. Many Deltaproteobacteria are anaerobic or microaerophilic. Additionally, some Epsilonproteobacteria were also found in the sediment samples and are known to be microaerophilic strains that could exploit the reduced sulphide produced by sulphate reducing Deltaproteobacteria. Our results based on high throughput amplicon sequencing are in line with the earlier studies from similar coastal sites from the Baltic sea surface sediments that have also found Proteobacteria, Bacteroidetes, Planctomycetes, Firmicutes, Acidobacteria, Chloroflexi and Cyanobacteria [56–59].

### Archaeal communities in sea water and sediment

The dominant archaeal phyla in the water was Thaumarchaeota whereas the communities in sediment samples were dominated by Bathyarchaeota and Euryarchaeota. Thaumarchaeota have been found to have an important role in the ammonia oxidation and nitrification in the Baltic Sea water [60, 61]. Almost all detected Thaumarchaeota belonged to the ammonia-oxidizing genus ‘*Candidatus Nitrosopumilus’*. In a recent study, [12] Halobacteria were found to dominate both in the sediment (31%) and water (38–58%) and Methanobacteria to be especially abundant in water (31–41%) samples from the Gulf of Finland. In addition, Thaumarchaeota were found from both sediment (14%) and water (8%) samples. This archaeal distribution is quite different from our results, yet it includes Thaumarchaeota both in water and sediment samples and Euryarchaeota in sediment samples which were the dominant phyla also in our study. The role of Archaea in sediment environments is much more studied than in water samples. Bathyarchaeota are known to participate in the cycling of carbon [62, 63] but also to participate in protein remineralisation [64] and they are ubiquitous in both marine and terrestrial anoxic sediments [65]. Euryarchaeota found from the sediment samples in our study were largely methanogenic Archaea belonging to Methanomicrobia class but also sequences from the order Thermoplasmatales, that are often found in sediment environments [66, 67], were detected. One explanation for the occurrence of these Thermoplasmatales could be the sulphide formation from the sulphate reduction that probably was ongoing as sulphate reducing Bacteria were found abundantly. Compte-Port et al. [67] found that Thermoplasmatales together with Bathyarchaeota were ubiquitous particularly in euxinic inland surface sediments compared to other anoxic sediments. In addition, co-occurrence of Bathyarchaeota and Thermoplasmata in both saline and freshwater sediments has been discovered also earlier but their relation of syntrophy or the utilisation of the same resources remains unclear [68]. The role of the detected Woesearchaeota that belong to a lineage of extremely reduced cell and genome size and lack the coding capacity for most amino acid biosynthetic pathways indicating dependence on other microorganisms for survival [62] is unknow. They may live in symbiotic or parasitic relationships with other microorganisms especially as they were detected from water with 0.2 μm pore size filters. A recent study by Rasigraf et al. [69] studied one sediment (depth 110 m) location from an open sea area in the Gulf of Bothnia. They found that Thaumarchaeota, Bathyarchaeota (formerly DSHVG-6), Euryarchaeota (ANME 2a-2b and Thermoplasmatales) and Pace/Woesearchaeota dominated the sediment surface (0–12.5 cm bsf). These same phyla were dominant in our study even though the study locations were much shallower (<20 m) and close to the coast line.

### Fungal communities in water and sediment

Ascomycota, Basiodiomycota and an unidentified fungus were abundant in water samples. In addition to these, the sediment samples also contained Chytridiomycota and Glomeromycota with higher relative abundance than in the water samples. Rozellomycota, also known as Cryptomycota, had high relative abundance only in Naantali PP sediment sample in contrast to other samples. Microbial Eukaryota have been detected in Baltic Sea water and sediments in many recent studies [52, 53, 69, 70]. However, they have mainly been removed from the analysis as they have not been specifically searched for or studied. Outside of the brackish environment, in the saline ocean environments, Ascomycota, Basidiomycota and Chytridiomycota are found to be the dominant planktonic fungal phyla [71, 72]. Cryptomycota have been frequently detected from freshwater and marine sediments and marine water [72–74]. Glomeromycota that were also frequently detected in all sediment samples is generally only a minor taxon in marine waters [72].

### Effects of extracellular DNA on the community composition

Marine sediments include both extra- and intracellular DNA. Part of the extracellular DNA is complexed to insoluble inorganic and organic matrices and aggregates and is not easily soluble and extractable without physical or chemical lysis [75, 76]. In this study, the DNase treatment method used before DNA extraction was targeted to remove the extracellular soluble DNA fraction from the sediment samples. The large share (>83%) of DNase removable soluble DNA in the sediment samples was in line with the results of Dell’Anno et al. [77], who showed that there was a large portion of removable extracellular DNA in the top sediment layer (63–82%). The composition and distribution of bacterial and archaeal phyla in DNase treated and untreated sediment samples were relatively similar. However, some differences especially in the fungal diversity was observed. This is very much in line with the findings by Lennon et al. [78], who reported that in general the extracellular DNA from different taxa degrades at a constant and equal rate and thus does not affect the overall detected microbial diversity. In cases where poorly adaptable taxa arrive outside the system, and die quickly in the new environment, the proportion of dying cells may expand the extracellular DNA pool and result in dissimilar community profiles compared to the indigenous population [78]. Fresh water sources with decaying organic matter that end up in sea water and sediment in coastal areas, especially in autumn time, may explain the difference in the DNA and DNase treated sediment pools of Fungi as Fungi are primary organic matter decayers.

### Environmental parameters and oil degradation potential

Environmental parameters, such as chloride, sulphate, nitrate, nitrite, and total phosphorus, in the water samples were mostly at the same concentration level in all studied samples. However, trends in environmental results could be seen between Naantali and Porvoo sites explaining part of the microbial diversity differences seen in the PcoA plots, as the two sites were separated probably based on petroleum hydrocarbon and PAH concentrations and salinity. In addition, the Naantali PP sample was also different form the Naantali 300 sample. This difference was probably due to higher concentration of total nitrogen and iron in the Naantali PP sample, but there were likely also other parameters affecting to these results, which were not measured in this study.

In case of sediment samples, higher PAH and organic matter concentrations separated Porvoo samples from the less contaminated Naantali samples. The fungal communities were more affected by the measured environmental parameters than the bacterial and archaeal communities as the reference site Porvoo B was separated from the more PAH compound and petroleum hydrocarbon contaminated Porvoo sites D and Q. Other environmental parameters (elemental concentrations of N, C, and S) were mostly at the same range in the samples and did not affect the microbial diversity in the studied sites.

The Porvoo site D contained ten to a hundred times more PAH-compounds (Table 3) than the other sediment samples. In comparison to all other sediment samples several bacterial, archaeal and fungal taxa were detected at statistically significantly higher relative abundance in amplicon sequencing in the site D. Bacterial taxon *Dehalococcoidia* (Napoli-4B-65) was found more abundantly in sediment D. Many species of the Dehalococcoidia class are regarded as organohalide respiring Bacteria [79]. However, not all of them seem to have reductive dehalogenase genes but some may have the ability to oxidise aromatic compounds [80]. In addition to PAH compounds, Porvoo sediments also contain relatively high amounts of different organohalides, such as octachlorodibenzofuran (OCDF) [81]. Acetothermia, three Bacteroidetes taxa and an Anaerolineaceae were found more abundantly from the sample D than from other samples. Acetothermia, a candidate phylum member, has been found from biogeochemical transformations in Alaska oil fields from produced deep waters [82]. The phylum Bacteroidetes has many known oil degraders and especially aromatic compound degraders [83, 84]. Representatives of Anaerolineaceae can anaerobically degrade n-alkanes and possibly also PAHs [85–87]. A characteristic of Anaerolineaceae is their association with *Methanosaeta*, Archaea that were also more abundantly detected in sediment D than from the other sediments. *Methanosaeta* and Anaerolineaceae have been suggested to syntrophically cooperate in methanogenic n-alkane degradation [85] and their coexistence has been discovered also in diesel-contaminated refuelling station soil [86]. Other Archaea that were more abundant in the sediment D than in the other sediments were several euryarchaeotal taxa including anaerobic methane oxidizers (ANME 2 and ANME 3), methanogenic Archaea (Methanomicrobiaceae, Methanosarcinaceae, ‘*Candidatus Methanofastidiosa*’) and Thermoplasmatales. Euryarchaeota and especially methanogenic Euryarchaeota have been found to participate in oil degradation in oil contaminated soils [66, 86, 88]. It was also noticed that in similar pristine soils their abundance was significantly lower [66, 86] indicating probable participation in oil degradation process.

Fungal taxa detected with statistically significantly higher relative abundance from the Porvoo site D than from the other sites included Glomeromycota. Glomeromycota are arbuscular mycorrhizal Fungi (AMF) that are obligatory plant root endosymbionts [89]. These AMF are considered to enhance the phytoremediation efficiency of soil hydrocarbon contaminants by enhancement of plant growth, increased biodegradative activity of roots and rhizosphere microorganisms and improved adsorption and bioaccumulation of hydrocarbons by roots [90]. However, Glomeromycota have been also found from submerged roots in aquatic environments and were regarded as root-associated Fungi (RAF) [91]. The role of Fungi in marine sediment including these AMF is largely unexplored. It is possible that they have some niche in marine sediment and especially in petroleum hydrocarbon or aromatic compound degradation with capabilities not yet known. The other fungal taxon more abundantly found in the PAH contaminated sediment was a *Caloplaca*, a lichen genus. No information on its role in marine sediment is available and hence its importance is also unknown. Many fungal strains are known to degrade oil and some of those like *Penicillium*, Pleosporaceae and *Mortierella* were detected with relatively low abundance in different sediment samples also in this study.

## Conclusions

Hypothesis of higher oil degradation potential in the long-term oil polluted coastal site microbiomes compared to nearby less exposed sites could not be confirmed. The number of genes participating in alkane degradation (*alk*B) or PAH-ring hydroxylation (PAH–RHDα) were detected in all water and sediment samples but no higher gene copy amounts were detected from the oil exposed sites. The oil degradation related gene copy numbers correlated with the number of 16S rRNA gene copies in sediment samples and not with the concentration of petroleum hydrocarbons or PAHs. In addition, bacterial, archaeal and fungal diversities were similar in oil exposed and less exposed sampling sites. This indicates overall, that both the clean and the more polluted sites at the Baltic Sea coastal areas had a potential for petroleum hydrocarbon degradation. Environmental parameters, including PAH, petroleum hydrocarbon and probably also other toxic compounds, seemed to affect the gene copy numbers of 16S rRNA genes of Bacteria and Archaea as well as the numbers of 5.8S rRNA genes of Fungi as these numbers were always lower in the near refinery sediment samples than in the less impacted reference sediment samples.

We showed for the first time that the active community of the coastal Baltic Sea water differed largely from the total community based on the analyses of amplicon sequencing of RNA and DNA fractions. The difference was most noticeable in the bacterial communities and also the distribution of dominant fungal phyla had noticeable differences indicating for example change in the community composition due to environmental or seasonal change whereas archaeal communities were more stable. The abundance, richness and diversity of Fungi was in general lower than that of Bacteria and Archaea. Furthermore, the sampling location influenced the fungal community composition, whereas the bacterial and archaeal communities were not influenced. This may indicate that fungal species that are adaptable to the Baltic Sea environments are few and that Fungi are potentially more vulnerable to or more affected by the environmental conditions than Bacteria and Archaea. The DNase treatment removed a large fraction of the extracellular soluble DNA from the sediment samples with only a slight effect on the detected microbial community profiles compared to untreated sediment samples.

## Supporting information

S1 Table Number of bacterial and archaeal 16S rRNA and fungal 5.8S rRNA gene copies from sea water. Copy numbers determined by qPCR from triplicate DNA extractions.
(PDF)

S2 Table Number of bacterial and archaeal 16S rRNA and fungal 5.8S rRNA gene copies. Copy numbers determined by qPCR from triplicate DNA extractions from untreated sediment or sediment treated with DNase.
(PDF)

S3 Table Number of PAH-RHDα Gram-negative (GN), Gram-positive (GP) and *alk*B genes copies in water samples. Copy numbers determined by qPCR from triplicate DNA and RNA sample extractions.
(PDF)

S4 Table Number of PAH-RHDα Gram-negative (GN), Gram-positive (GP), *alk*B and bacterial 16S rRNA genes copies in sediment or sediment treated with DNase. Copy numbers determined by qPCR from triplicate DNA extractions.
(PDF)

S5 Table Amount of extracted DNA from untreated and DNase-treated sediment samples. On the right percent (%) of DNA left after DNase treatment compared to untreated DNA extractions, analysed from triplicate samples.
(PDF)

S6 Table Average number of identified sequence reads, identified OTUs, estimated OTU richness (Chao1) and Shannon diversity index calculated for the bacterial, archaeal and fungal communities. (PDF)

S1 Fig Principal coordinates analysis of bacterial sequences from water and sediment samples. (TIFF)

S2 Fig Principal coordinate analysis of combined bacterial, archaeal and fungal community composition and environmental parameters from RNA and DNA isolated from the sea water samples. All presented environmental variables were statistically significant (p<0.05) with 999 permutations.
(TIFF)

S3 Fig Principal coordinate analysis of bacterial community composition and environmental parameters from RNA and DNA isolated from the sea water samples. All presented environmental variables were statistically significant (p<0.05) with 999 permutations.
(TIFF)

S4 Fig Principal coordinate analysis of fungal community composition and environmental parameters from sediment samples. All presented environmental variables were statistically significant (p<0.05) with 999 permutations.
(TIFF)
