# Supplementary material for: Oil degradation potential of microbial communities in water and sediment of Baltic Sea coastal area
Source: PLoS One. 2019 Jul 2;14(7):e0218834. doi: 10.1371/journal.pone.0218834 (PMC6605675; doi:10.1371/journal.pone.0218834)
Supplement: S1 Table — Copy numbers determined by qPCR from triplicate DNA extractions. (PDF) [file pone.0218834.s001.pdf]

S1 Table. Number of bacterial and archaeal 16S rRNA and fungal 5.8S rRNA gene copies from sea water. Copy numbers determined by qPCR from triplicate DNA extractions.

Site	Bacteria		Archaea		Fungi	
	Copy number mL^{-1}	SD	Copy number mL^{-1}	SD	Copy number mL^{-1}	SD
Porvoo Q	1.13E+07	1.22E+06	4.18E+05	3.13E+04	3.61E+03	1.18E+03
Porvoo D	1.23E+07	2.63E+06	3.96E+05	4.80E+04	2.16E+03	1.72E+03
Porvoo B	1.18E+07	6.48E+06	3.99E+05	2.62E+05	7.23E+03	4.86E+03
Naantali PP	2.05E+07	2.67E+06	9.66E+05	1.99E+05	8.49E+03	9.17E+02
Naantali 300	2.36E+07	6.12E+06	9.04E+05	6.99E+04	2.23E+04	6.77E+02
