# Supplementary material for: Oil degradation potential of microbial communities in water and sediment of Baltic Sea coastal area
Source: PLoS One. 2019 Jul 2;14(7):e0218834. doi: 10.1371/journal.pone.0218834 (PMC6605675; doi:10.1371/journal.pone.0218834)
Supplement: S2 Table — Copy numbers determined by qPCR from triplicate DNA extractions from untreated sediment or sediment treated with DNase. (PDF) [file pone.0218834.s002.pdf]

S2 Table. Number of bacterial and archaeal 16S rRNA and fungal 5.8S rRNA gene copies.

Copy numbers determined by qPCR from triplicate DNA extractions from untreated sediment or sediment treated with DNase.

Site	Bacteria sediment		Bacteria DNase treated sediment		Archaea sediment		Archaea DNase treated sediment		Fungi sediment		Fungi DNase treated sediment	
	Copy number $g^{-1}dw$	SD	Copy number $g^{-1}dw$	SD	Copy number $g^{-1}dw$	SD	Copy number $g^{-1}dw$	SD	Copy number $g^{-1}dw$	SD	Copy number $g^{-1}dw$	SD
Porvoo Q	2.67E+10	3.28E+09	2.23E+09	1.24E+09	9.56E+08	1.77E+08	7.83E+07	4.33E+07	1.40E+07	4.66E+06	1.76E+06	6.65E+05
Porvoo D	2.15E+10	8.81E+09	2.04E+09	7.19E+08	1.49E+09	5.53E+08	1.42E+08	2.21E+07	4.38E+06	1.98E+06	1.97E+06	7.17E+05
Porvoo B	5.84E+10	8.31E+09	1.03E+10	4.65E+09	3.34E+09	1.08E+08	1.77E+08	6.08E+07	4.93E+07	6.61E+06	1.38E+07	5.00E+06
Naantali PP	3.86E+09	3.18E+09	4.21E+07	2.20E+07	2.24E+08	2.03E+08	3.69E+06	1.48E+06	1.04E+06	7.54E+05	< 1.0E+06	
Naantali 300	5.61E+09	2.93E+08	3.43E+08	3.69E+07	2.56E+08	2.35E+07	9.20E+06	1.93E+06	5.64E+06	1.38E+06	< 1.0E+06	
