# Supplementary material for: Oil degradation potential of microbial communities in water and sediment of Baltic Sea coastal area
Source: PLoS One. 2019 Jul 2;14(7):e0218834. doi: 10.1371/journal.pone.0218834 (PMC6605675; doi:10.1371/journal.pone.0218834)
Supplement: S3 Table — Copy numbers determined by qPCR from triplicate DNA and RNA sample extractions. (PDF) [file pone.0218834.s003.pdf]

S3 Table. Number of PAH-RHD$_\alpha$ Gram-negative (GN), Gram-positive (GP) and *alk*B genes copies in water samples.
Copy numbers determined by qPCR from triplicate DNA and RNA sample extractions.

Site	GN, DNA		GN, RNA		GP, DNA		GP, RNA		*alk*B, DNA		*alk*B, RNA	
	Copy number mL^{-1}	SD	Copy number mL^{-1}	SD	Copy number mL^{-1}	SD	Copy number mL^{-1}	SD	Copy number mL^{-1}	SD	Copy number mL^{-1}	SD
Porvoo Q	2.07E+04	3.85E+03	<5.0E+01		4.20E+04	5.43E+03	1.24E+02	1.89E+01	1.15E+04	1.07E+03	1.11E+02	1.97E+01
Porvoo D	1.54E+04	2.12E+03	<5.0E+01		3.96E+04	1.68E+04	< 4.0E+01		9.91E+03	1.27E+03	< 4.0E+01	
Porvoo B	1.50E+04	1.06E+04	<5.0E+01		2.89E+04	1.32E+04	< 4.0E+01		7.46E+03	2.57E+03	< 4.0E+01	
Naantali PP	5.53E+04	3.70E+04	<5.0E+01		5.76E+04	4.65E+03	< 4.0E+01		2.05E+04	5.54E+03	< 4.0E+01	
Naantali 300	3.54E+05	4.22E+05	<5.0E+01		5.61E+04	2.80E+04	8.80E+01	1.18E+01	1.85E+04	6.78E+03	3.15E+02	1.69E+01
