# Supplementary material for: Oil degradation potential of microbial communities in water and sediment of Baltic Sea coastal area
Source: PLoS One. 2019 Jul 2;14(7):e0218834. doi: 10.1371/journal.pone.0218834 (PMC6605675; doi:10.1371/journal.pone.0218834)
Supplement: S4 Table — Copy numbers determined by qPCR from triplicate DNA extractions. (PDF) [file pone.0218834.s004.pdf]

S4 Table. Number of PAH-RHD$_{\alpha}$ Gram-negative (GN), Gram-positive (GP), *alk*B and bacterial 16S rRNA genes copies in sediment or sediment treated with DNase.

Copy numbers determined by qPCR from triplicate DNA extractions.

Site	GN, sediment		GN, DNase treated sediment		GP, sediment		GP, DNase treated sediment		*alk*B, sediment		*alk*B, DNase treated sediment	
	Copy number g^{-1}dw	SD	Copy number g^{-1}dw	SD	Copy number g^{-1}dw	SD	Copy number g^{-1}dw	SD	Copy number g^{-1}dw	SD	Copy number g^{-1}dw	SD
Porvoo Q	1.72E+06	2.96E+05	2.63E+05	1.53E+05	2.36E+06	7.78E+05	3.47E+05	1.92E+05	1.07E+07	1.11E+06	9.95E+05	7.00E+05
Porvoo D	1.06E+06	2.34E+05	2.35E+05	5.29E+04	2.60E+06	1.42E+06	3.45E+05	2.55E+04	6.47E+06	2.30E+06	7.11E+05	2.62E+05
Porvoo B	1.21E+06	2.66E+05	9.69E+05	3.25E+05	2.87E+06	1.29E+06	1.31E+06	6.11E+05	4.34E+07	8.50E+06	3.96E+06	2.26E+06
Naantali PP	3.86E+05	2.06E+05	7.30E+03	2.72E+03	6.12E+05	4.65E+05	8.83E+03	7.85E+03	1.88E+06	1.71E+06	2.16E+04	1.54E+04
Naantali 300	6.95E+05	9.15E+04	3.66E+04	6.67E+03	7.70E+05	2.23E+05	6.57E+04	1.72E+04	2.70E+06	3.92E+05	1.89E+05	5.03E+03
