# Supplementary material for: Oil degradation potential of microbial communities in water and sediment of Baltic Sea coastal area
Source: PLoS One. 2019 Jul 2;14(7):e0218834. doi: 10.1371/journal.pone.0218834 (PMC6605675; doi:10.1371/journal.pone.0218834)
Supplement: S5 Table — On the right percent (%) of DNA left after DNase treatment compared to untreated DNA extractions, analysed from triplicate samples. (PDF) [file pone.0218834.s005.pdf]

S5 Table. Amount of extracted DNA from untreated and DNase-treated sediment samples. On the right percent (%) of DNA left after DNase treatment compared to untreated DNA extractions, analysed from triplicate samples.

Site	DNA ng g^{-1} dw	DNA after DNase treatment ng g^{-1} dw	% of DNase treated DNA from untreated DNA
Porvoo Q	8.6	0.6	7.2
	10.5	0.6	5.4
	10.4	1.4	13.4
Porvoo D	17.2	1.4	8.1
	10.5	1.5	14.3
	12.2	2.0	16.3
Porvoo B	19.4	1.9	9.6
	20.3	1.9	9.2
	19.4	0.8	4.3
Naantali PP	5.9	0.05	0.8
	2.5	0.07	2.9
	0.9	0	0.0
Naatali 300	4.1	0.1	3.2
	3.2	0.2	5.5
	3.6	0.2	4.7
