# Supplementary material for: Oil degradation potential of microbial communities in water and sediment of Baltic Sea coastal area
Source: PLoS One. 2019 Jul 2;14(7):e0218834. doi: 10.1371/journal.pone.0218834 (PMC6605675; doi:10.1371/journal.pone.0218834)
Supplement: S6 Table — (PDF) [file pone.0218834.s006.pdf]

S6 Table. Average number of identified sequence reads, identified OTUs, estimated OTU richness (Chao1) and Shannon diversity index calculated for the bacterial, archaeal and fungal communities.

	Sample type	Number of sequences		Number of OTUs		Chao1		Shannon	
**Bacteria**									
Naantali 300	Water RNA	5686	±6370	3056	±3077	9958	±9583	10.3	±1.0
	Water DNA	3046	±1790	1655	±797	5707	±3380	9.7	±0.4
	Sediment	3184	±2745	1869	±1608	6236	±5380	8.3	±4.1
	Sediment DNase	2931	±1139	1374	±410	4186	±1094	9.4	±0.3
Naantali PP	Water RNA	6067	±5057	3728	±2834	13328	±8841	10.7	±1.2
	Water DNA	2729	±506	1620	±302	5678	±1379	9.7	±0.4
	Sediment	9379	±8822	4214	±3177	11486	±7262	10.8	±0.7
	Sediment DNase	2011	±1423	978	±603	2625	±1197	9.0	±0.9
Porvoo B	Water RNA	2394	±398	1617	±196	5138	±200	10.2	±0.1
	Water DNA	2134	±165	1168	±151	3803	±400	9.2	±0.4
	Sediment	14370	±1614	6890	±796	24688	±3134	11.1	±0.1
	Sediment DNase	6013	±875	2501	±284	9852	±1508	9.5	±0.1
Porvoo D	Water RNA	2100	±339	1488	±212	5424	±690	10.1	±0.2
	Water DNA	2331	±680	1419	±315	4704	±808	9.8	±0.2
	Sediment	5592	±1597	3241	±718	10270	±2261	10.9	±0.2
	Sediment DNase	6300	±2096	2980	±875	9582	±2460	10.3	±0.3
Porvoo Q	Water RNA	2308	±347	1632	±148	5911	±633	10.2	±0.1
	Water DNA	2278	±813	1403	±478	4781	±1721	9.7	±0.4
	Sediment	4847	±2236	2634	±1124	8070	±3145	10.5	±0.6
	Sediment DNase	6070	±386	2774	±201	8871	±1265	10.2	±0.1
**Archaea**									
Naantali 300	Water RNA	10205	±4312	3039	±867	6471	±1695	10.1	±0.2
	Water DNA	1723	±118	1044	±64	2778	±276	9.4	±0.1
	Sediment	24	±13						
	Sediment DNase	4	±2						
Naantali PP	Water RNA	14639	±6603	3857	±1171	8115	±2297	10.2	±0.2
	Water DNA	3076	±327	1550	±143	3908	±435	9.7	±0.1
	Sediment	6716	±4591	1534	±847	3068	±1411	8.4	±0.5
	Sediment DNase	45	±33	28	±14	62	±21	4.4	±0.5
Porvoo B	Water RNA	6002	±377	2059	±47	4335	±147	9.8	±0.0
	Water DNA	1260	±149	747	±68	2068	±193	8.9	±0.1
	Sediment	29	±11						
	Sediment DNase	6	±3						
Porvoo D	Water RNA	7663	±2236	2552	±475	5707	±640	10.0	±0.1
	Water DNA	2344	±407	1214	±134	3164	±305	9.4	±0.1
	Sediment	17158	±2959	3740	±379	7211	±700	9.3	±0.2
	Sediment DNase	3471	±834	733	±120	1648	±130	6.8	±0.1
Porvoo Q	Water RNA	6916	±1545	2341	±331	5406	±498	9.9	±0.1
	Water DNA	2377	±660	1193	±256	3246	±697	9.3	±0.2
	Sediment	9071	±3384	2139	±608	4301	±1010	8.6	±0.3
	Sediment DNase	1203	±1233	306	±241	732	±455	6.3	±0.5

	Sample type	Number of sequences		Number of OTUs		Chao1		Shannon	
**Fungi**									
Naantali 300	Water RNA	4887	±684	991	±53	2126	±153	7.5	±0.0
	Water DNA	3241	±321	851	±72	1832	±213	7.7	±0.2
	Sediment	2335	±275	465	±23	947	±87	7.0	±0.1
	Sediment DNase	1417	±256	169	±19	364	±41	5.5	±0.2
Naantali PP	Water RNA	5614	±872	920	±86	1982	±244	6.6	±0.3
	Water DNA	12316	±2813	1989	±361	3975	±719	8.2	±0.3
	Sediment	3516	±1209	424	±125	858	±237	6.3	±0.1
	Sediment DNase	2496	±964	143	±48	337	±88	3.7	±0.6
Porvoo B	Water RNA	3988	±665	1061	±143	2265	±395	8.0	±0.1
	Water DNA	2126	±386	410	±11	968	±114	4.7	±0.4
	Sediment	1948	±102	522	±17	1495	±284	6.6	±0.1
	Sediment DNase	1322	±191	379	±7	925	±61	6.7	±0.1
Porvoo D	Water RNA	3623	±609	969	±92	2150	±244	8.0	±0.0
	Water DNA	2318	±129	519	±39	1355	±76	5.5	±0.5
	Sediment	6528	±286	1030	±102	2341	±329	6.9	±0.6
	Sediment DNase	4337	±1046	758	±59	1776	±184	7.0	±0.3
Porvoo Q	Water RNA	5086	±623	1247	±114	2736	±254	8.2	±0.2
	Water DNA	2690	±569	520	±88	1219	±247	5.1	±0.3
	Sediment	12443	±12919	1673	±1215	3560	±2135	7.5	±0.3
	Sediment DNase	3446	±367	685	±60	1695	±94	6.7	±0.5

Site	DNA (ng g^{-1} dw)	DNA after DNase treatment (ng g^{-1} dw)	DNA left after DNase treatment (%)
**Porvoo Q**	8.6	0.6	7.2
	10.5	0.6	5.4
	10.4	1.4	13.4
**Porvoo D**	17.2	1.4	8.1
	10.5	1.5	14.3
	12.2	2.0	16.3
**Porvoo B**	19.4	1.9	9.6
	20.3	1.9	9.2
	19.4	0.8	4.3
**Naantali PP**	5.9	0.05	0.8
	2.5	0.07	2.9
	0.9	0	0.0
**Naatali 300**	4.1	0.1	3.2
	3.2	0.2	5.5
	3.6	0.2	4.7
