# Supplementary figures and images for: Oil degradation potential of microbial communities in water and sediment of Baltic Sea coastal area
Source: PLoS One. 2019 Jul 2;14(7):e0218834. doi: 10.1371/journal.pone.0218834 (PMC6605675; doi:10.1371/journal.pone.0218834)

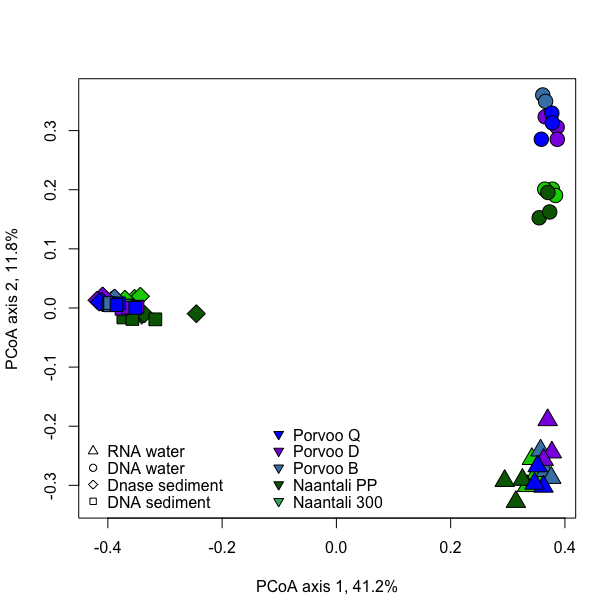

Supplement: S1 Fig — (TIFF) [file pone.0218834.s007.tiff]

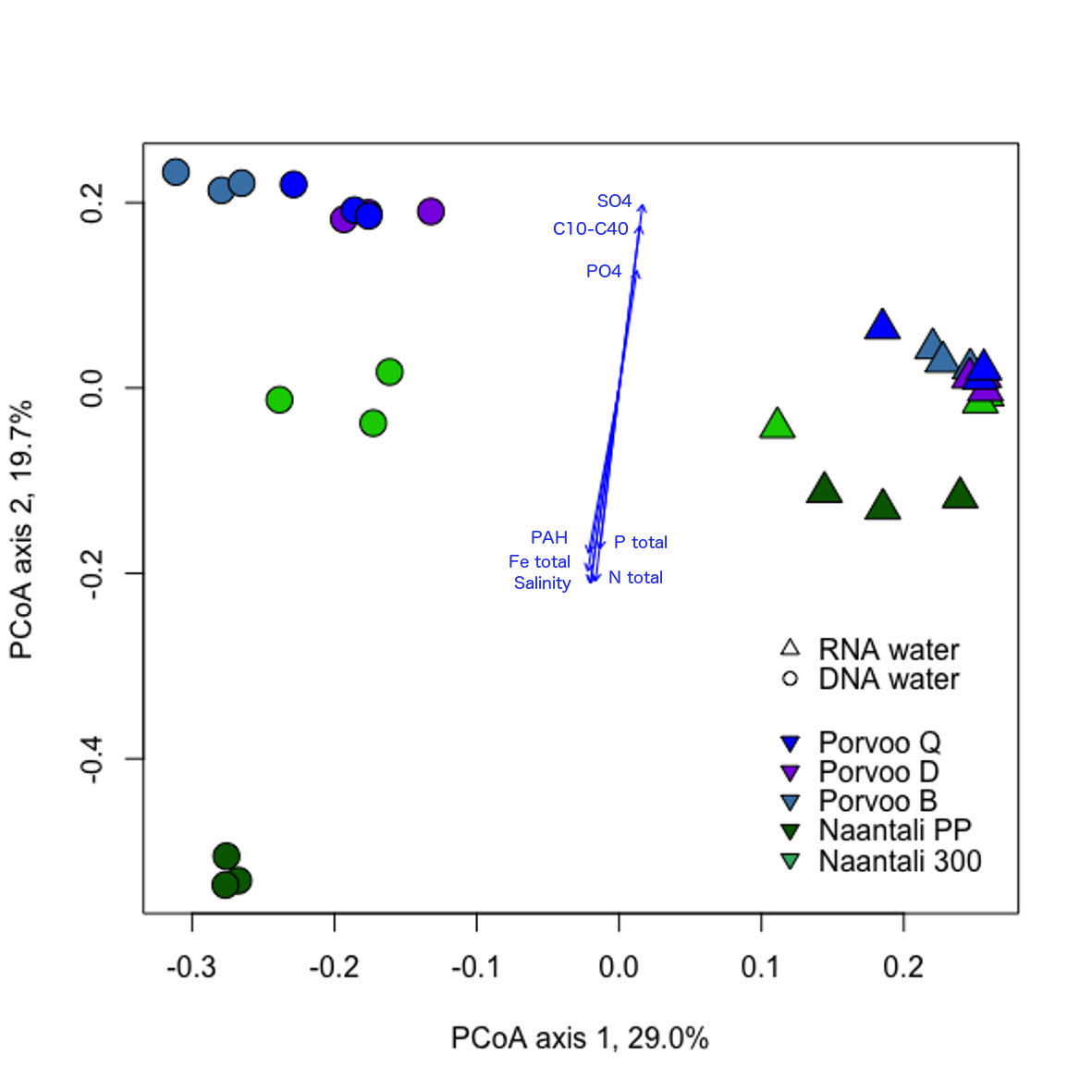

Supplement: S2 Fig — All presented environmental variables were statistically significant (p<0.05) with 999 permutations. (TIFF) [file pone.0218834.s008.tiff]

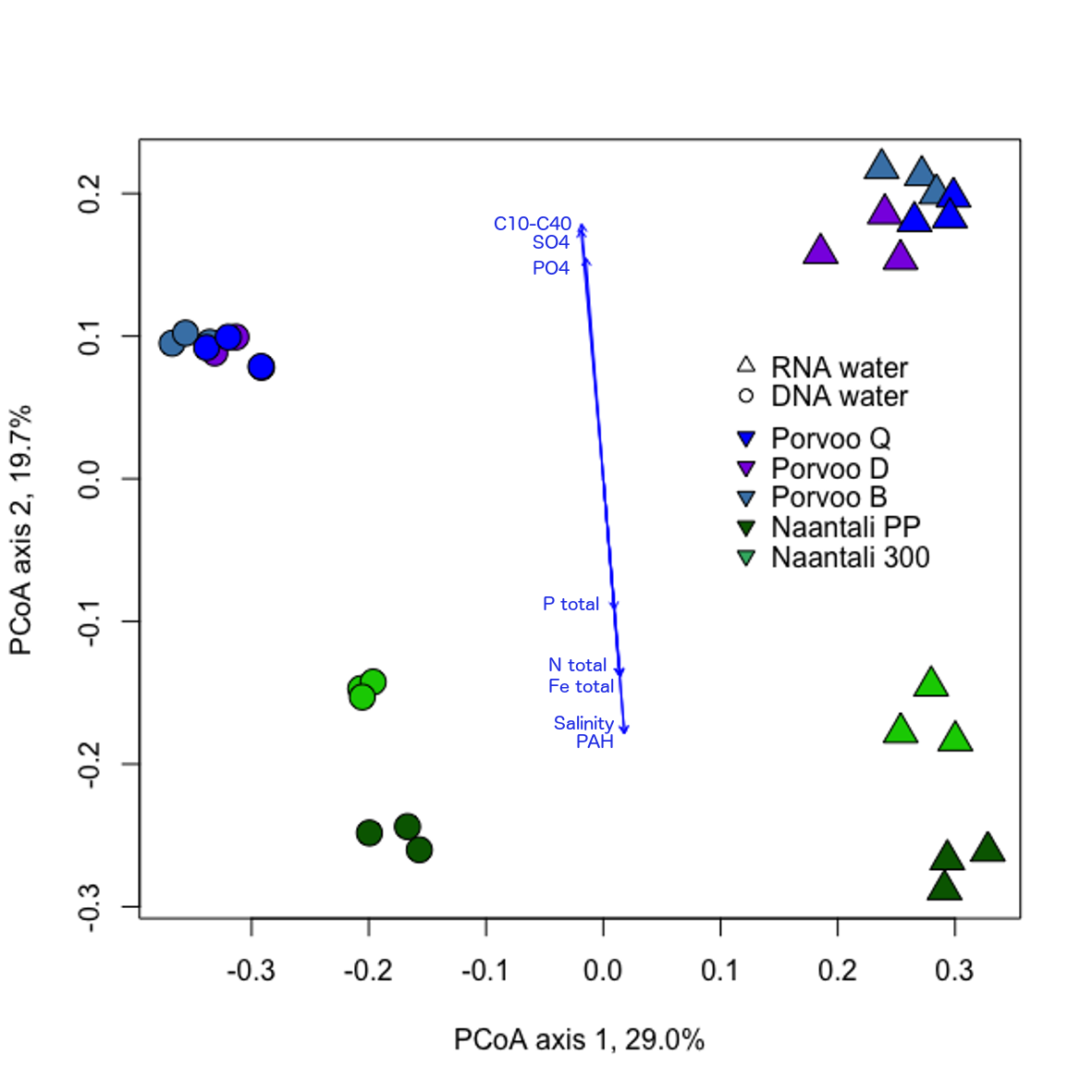

Supplement: S3 Fig — All presented environmental variables were statistically significant (p<0.05) with 999 permutations. (TIFF) [file pone.0218834.s009.tiff]

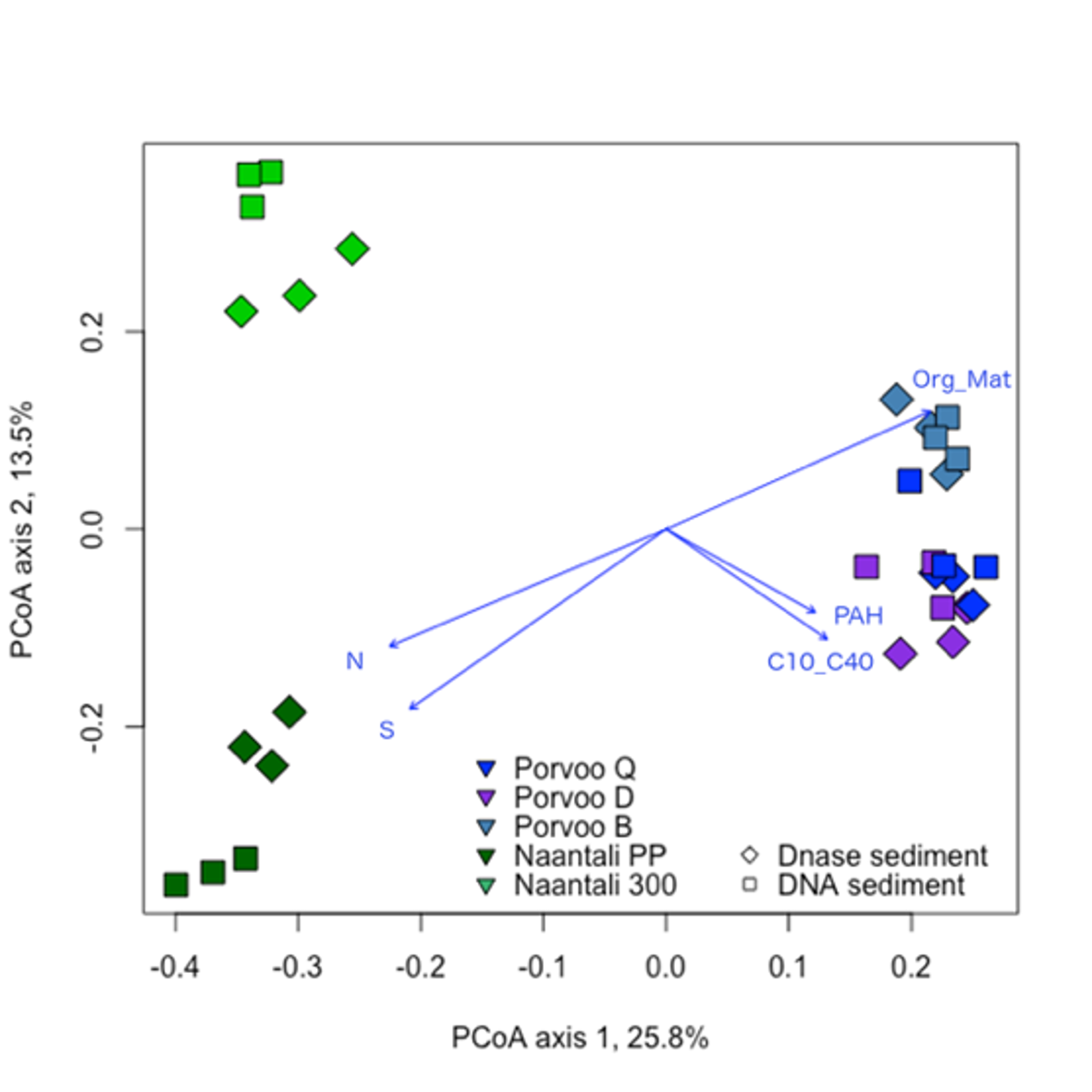

Supplement: S4 Fig — All presented environmental variables were statistically significant (p<0.05) with 999 permutations. (TIFF) [file pone.0218834.s010.tiff]
